# Modifying Alzheimer’s disease pathophysiology with photobiomodulation: model, evidence, and future with EEG-guided intervention

**DOI:** 10.3389/fneur.2024.1407785

**Published:** 2024-08-23

**Authors:** Lew Lim

**Affiliations:** Vielight Inc., Toronto, ON, Canada

**Keywords:** photobiomodulation, Alzheimer’s disease, pathophysiology, clinical evidence, clinical studies, EEG, artificial intelligence, default mode network

## Abstract

This manuscript outlines a model of Alzheimer’s Disease (AD) pathophysiology in progressive layers, from its genesis to the development of biomarkers and then to symptom expression. Genetic predispositions are the major factor that leads to mitochondrial dysfunction and subsequent amyloid and tau protein accumulation, which have been identified as hallmarks of AD. Extending beyond these accumulations, we explore a broader spectrum of pathophysiological aspects, including the blood–brain barrier, blood flow, vascular health, gut-brain microbiodata, glymphatic flow, metabolic syndrome, energy deficit, oxidative stress, calcium overload, inflammation, neuronal and synaptic loss, brain matter atrophy, and reduced growth factors. Photobiomodulation (PBM), which delivers near-infrared light to selected brain regions using portable devices, is introduced as a therapeutic approach. PBM has the potential to address each of these pathophysiological aspects, with data provided by various studies. They provide mechanistic support for largely small published clinical studies that demonstrate improvements in memory and cognition. They inform of PBM’s potential to treat AD pending validation by large randomized controlled studies. The presentation of brain network and waveform changes on electroencephalography (EEG) provide the opportunity to use these data as a guide for the application of various PBM parameters to improve outcomes. These parameters include wavelength, power density, treatment duration, LED positioning, and pulse frequency. Pulsing at specific frequencies has been found to influence the expression of waveforms and modifications of brain networks. The expression stems from the modulation of cellular and protein structures as revealed in recent studies. These findings provide an EEG-based guide for the use of artificial intelligence to personalize AD treatment through EEG data feedback.

## Introduction

Alzheimer’s disease (AD) is a major public health problem all over the world. Therapeutic strategies have been explored for several decades, but no curative treatment has been developed (1). Although the FDA has recently approved lecanemab (Leqembi) and donanemab (Kisunla) for treating cognitive decline in early Alzheimer’s disease, their efficacy is modest, with potential side effects like amyloid-related imaging abnormalities (ARIA), and their long-term impact on disease progression remains uncertain, particularly in advanced stages (2, 3). However, these drugs represent a significant advancement by objectively reducing amyloid beta (Aβ) markers, which purportedly slow disease progression in patients with early-stage AD, marking an important first step toward more effective therapies.

AD pathophysiology is a complex and well-researched landscape without clear solutions at the end. While the research for clear answers continues, we will review the pathophysiological aspects revealed in AD research, synthesize relevant photobiomodulation (PBM) research findings demonstrating its ability to modify these aspects, and then evaluate PBM as a potential candidate therapy. While the mechanisms form much of the discussion, we place considerable value on the effects on humans in clinical studies, which will also be reviewed for the impact of various parameters of PBM. The clinical studies reviewed are available as peer-reviewed publications up to January 31, 2024.

## Methodology

This section outlines a comprehensive methodology for reviewing and analyzing the effects of PBM on AD pathophysiology. The study structure is designed to systematically explore the potential of PBM to address the complex underlying pathophysiology of AD even though the device user interface can be simple. It also considers the future directions for personalized interventions for better outcomes. With these goals, the manuscript is organized into several key components:

**Introduction to PBM mechanisms:** This section explains the fundamental mechanisms of PBM relevant to AD therapy. It introduces the concept of using brain waveforms and networks.

**Pathophysiological modeling:** This extensive section provides a detailed analysis of the impact of PBM on AD pathophysiology, synthesizing the two areas to understand potential therapeutic effects.

**AD symptom Analysis:** The analysis extends to reviews of AD symptoms, with a focus on precursor symptoms that are responsive to PBM. This includes an examination of brain networks, connectivity, and waveforms, expressed in electroencephalography (EEG) data, to identify potential targets for intervention.

**Clinical studies review:** A systematic review and analysis of available clinical studies on PBM’s outcomes in AD is conducted to gain insights into effective parameters.

**Parameter analysis:** Various PBM parameters are evaluated to determine their impact on AD. This analysis translates the findings into practical applications for PBM devices, with a focus on wavelength, pulse rate, and light source positioning.

**Future directions:** The final section explores the potential of using EEG-guided pathophysiological modifications and clinical outcomes in AD treatment. It also considers the incorporation of artificial intelligence (AI) to personalize PBM treatments.

**Figure 1:**
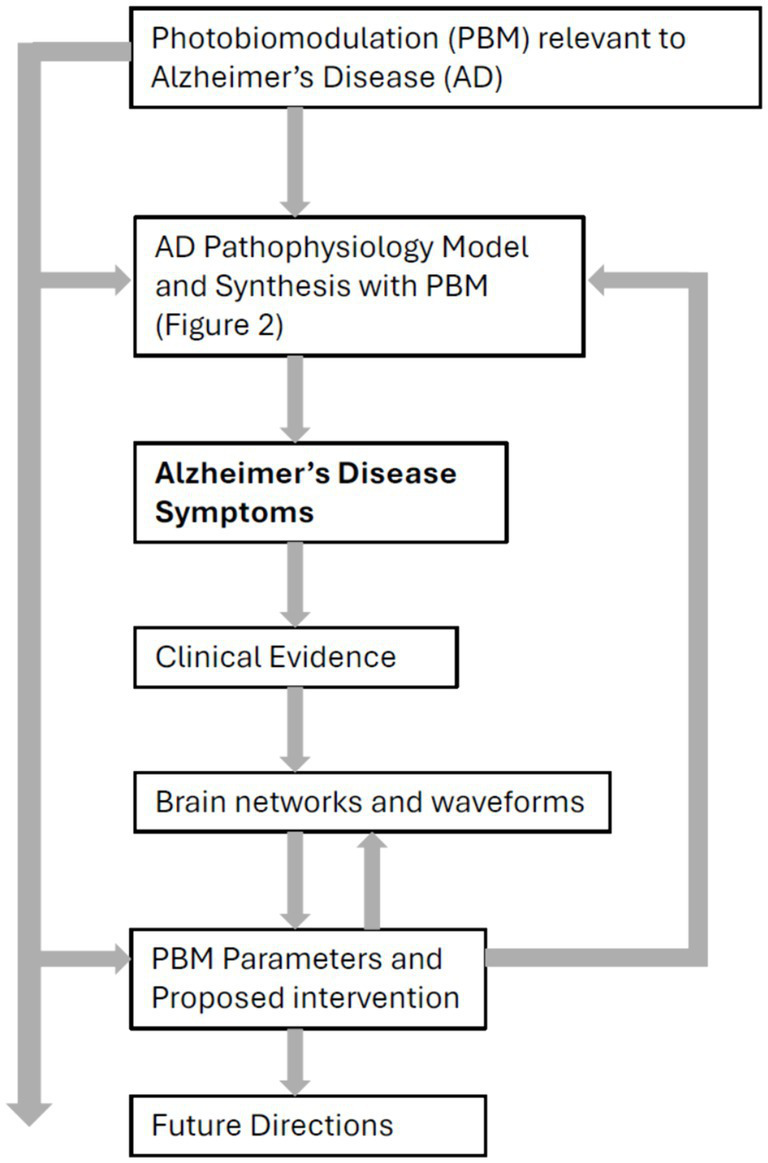
Schematic structure of the manuscript.

The manuscript acknowledges that current data may not be sufficient for definitive therapeutic strategies, but it aims to guide future investigations for improved outcomes. By synthesizing pathophysiological findings with brain waveform and network expressions, the study identifies potential targets for transcranial and intranasal PBM interventions. The approach is primarily based on literature reviews, incorporating analyses and forward-looking research and technology development strategies. The structure of the manuscript is illustrated in Figure 1, reflecting the methodology presented here.

## Results

### Relevance of photobiomodulation to Alzheimer’s disease treatment

PBM employs commercially available non-thermal device-based techniques that utilize light from LEDs or lasers in the red to near-infrared (NIR) spectrum. This light stimulates cellular processes, notably in mitochondria, enhancing energy production and initiating a cascade of biological responses important for cellular health. PBM’s effects are primarily attributed to its stimulation of cytochrome c oxidase (CcO) within the mitochondrial electron transport chain and activation of heat/light-gated ion channels. These actions facilitate increased ATP synthesis, nitric oxide (NO) release, reduced oxidative stress, and altered gene transcription factors, although the full scope of the mechanisms remains unclear. The therapeutic potential of PBM in AD lies in its multifaceted approach to disease pathology modulation. By influencing mitochondrial function, PBM can directly affect the neurodegenerative processes central to AD. Additionally, its role in modulating inflammatory responses, encouraging cell proliferation, and enhancing cellular signaling pathways positions PBM as a promising therapeutic option for AD (4–6).

Brain PBM devices can be marketed in the U.S. as low-risk general wellness products for mental acuity without FDA clearance, but specific medical claims require official FDA approval (7).

**Figure 2:**
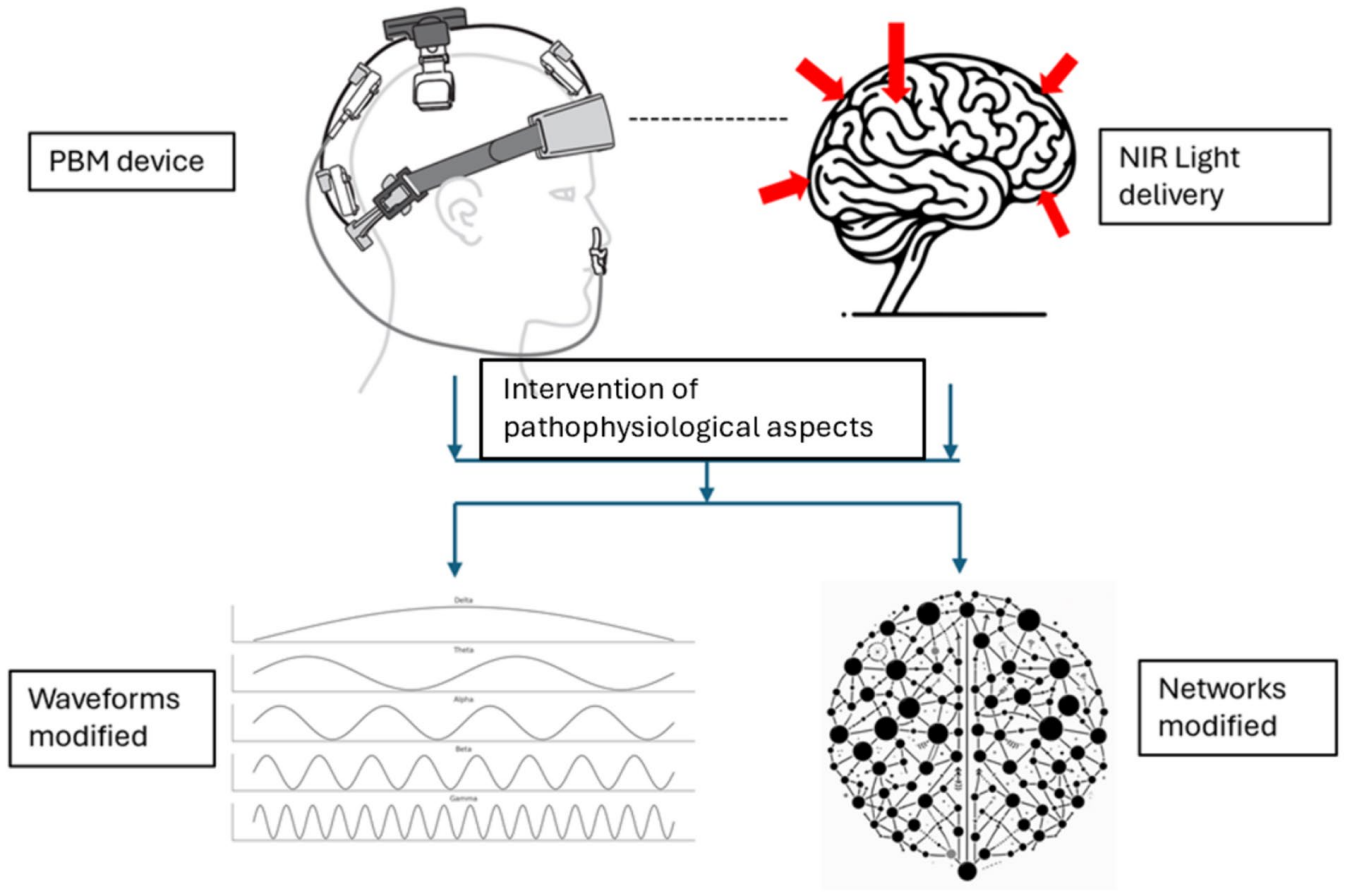
Modification of brain waveforms/oscillations and networks resulting from the intervention of pathophysiological aspects with PBM (Device rendering obtained from courtesy of Vielight Inc).

The subsequent sections will elucidate how these mechanisms translate to observable changes in biomarkers and brain function. Changes in brain function are represented by the restoration of disrupted brain waveforms/oscillations and networks in AD, as illustrated in Figure 2.

PBM’s versatility as a single intervention that has not been associated with major side effects underscores its potential as a safe neurological therapy for traumatic brain injury as an example. The capacity for personalization and adaptability to continuous experimentation further enhances its appeal for addressing the complex pathology of AD (8).

### An Alzheimer’s disease pathophysiology model and its synthesis with photobiomodulation

**Figure 3:**
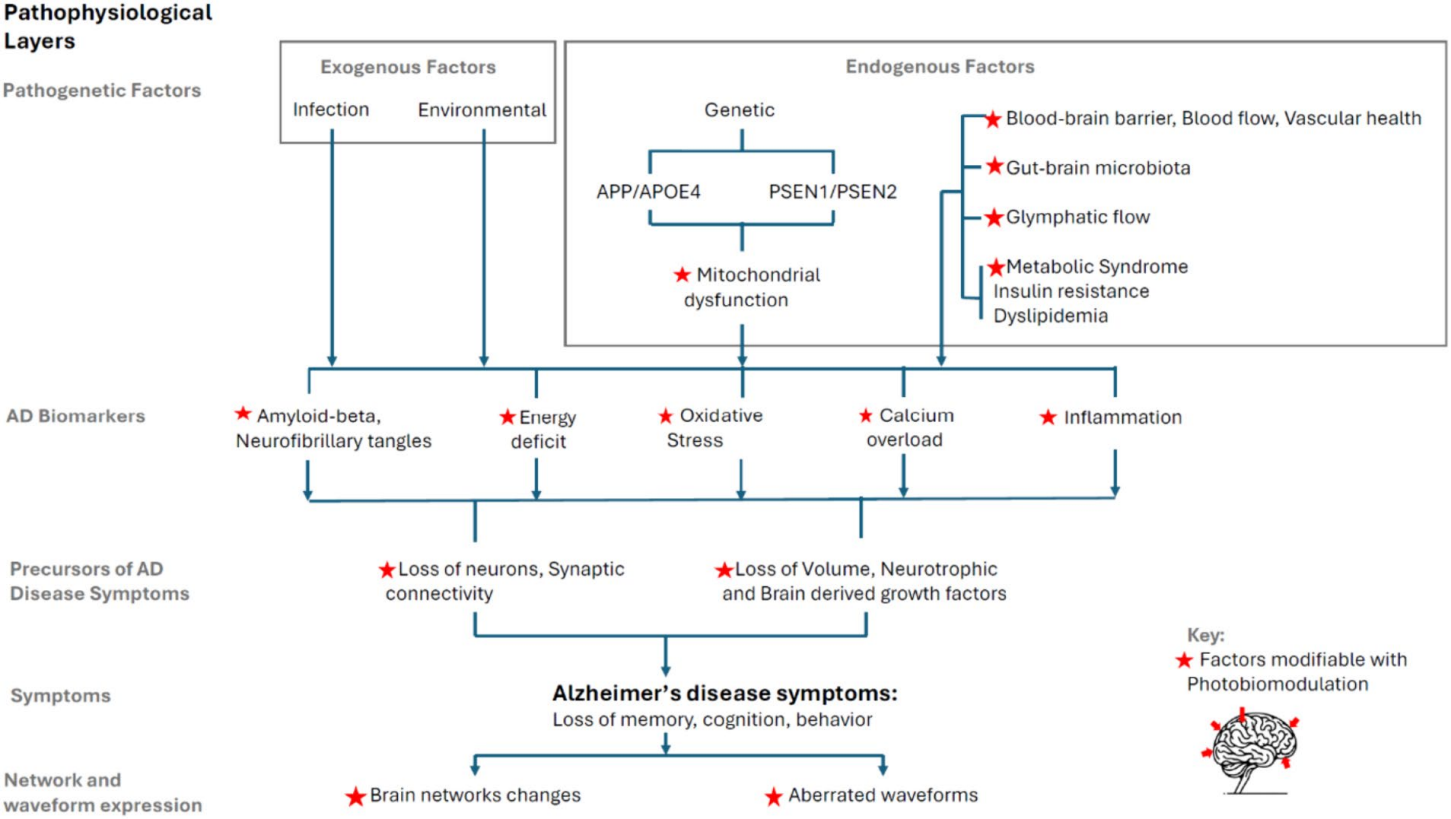
Layered AD pathophysiology model and synthesis with PBM.

The pathophysiological model of AD presented here is intended to be comprehensive in breadth, showing the progression from genesis to symptoms in progressive layers. Figure 3 schematically presents the progression, drawing on existing literature.

AD pathophysiology extends beyond the well-documented Aβ plaques and neurofibrillary tangles (NFTs) to encompass a range of exogenous factors (environmental factors, infections) and endogenous factors (genetic predispositions, blood–brain barrier integrity, cerebral blood flow, vascular health, gut-brain axis and microbiota, glymphatic system, metabolic syndrome, insulin resistance and dyslipidemia).

A significant aspect of AD pathology includes mitochondrial dysfunction, which is implicated in Aβ aggregation, NFT formation, neuroinflammation, oxidative stress, calcium dysregulation, and reduced energy production and metabolism.

The model categorizes the progression of AD into pathogenesis, highlighting the role of both exogenous and endogenous factors; mitochondrial dysfunction as a critical underpinning of disease progression; the precursors to AD symptoms, such as loss of neurons and synaptic connectivity, volume, and neurotrophic growth factors; and the expression of AD symptoms, notably cognitive decline and behavioral changes.

Recent discoveries have shown that AD affects brain networks and waveforms, making these expressions convenient avenues to guide PBM interventions.

In summary, this model delineates the complex interplay among factors contributing to AD and illustrates how diverse PBM mechanisms of action offer promising pathways for intervention. By targeting many of these pathophysiological factors, PBM presents a multifaceted approach to mitigating AD progression.

The various pathophysiological factors in the model, under different progressive layers are expounded in the sections below.

### Factors affecting Alzheimer’s disease pathogenesis

The genesis of AD pathology is influenced by genetic factors, environmental factors, infections, blood–brain barrier integrity, cerebral blood flow, and vascular health.

### Genetic predisposition

It can be argued that the genesis or origin of AD pathology is characterized by a complex interplay of genetic and environmental factors, which makes its onset challenging to predict. Key genes implicated in AD include amyloid precursor protein (APP), presenilin 1 (PSEN1), presenilin 2 (PSEN2), and apolipoprotein E (APOE) (9). Notably, the APOE gene’s ε4 allele is a significant risk factor for late-onset AD (10) and is associated with an elevated risk of cerebral amyloid angiopathy and age-related cognitive decline, even during the normal aging process (11). Conversely, the ε2 allele is associated with a reduced risk of AD. This intricate genetic landscape presents considerable hurdles in the development of effective APOE-targeted therapies, with no successful long-term interventions to date (12).

However, mutations in mitochondrial DNA (mtDNA), whether inherited or accumulated over time, can lead to mitochondrial damage, compromising their function and ultimately resulting in cell death and disease (13). Mutations in mtDNA can cause cognitive deficits like those seen in AD, highlighting the importance of mtDNA in proper cognitive function (13). Additionally, increased oxidative damage to mtDNA, due to its proximity to reactive oxygen species (ROS) generation, can give rise to mutations that affect mitochondrial function and their abundance (14). Therefore, mutations in mtDNA can disrupt mitochondrial bioenergetics, leading to energy deficits in neurons and contributing to the pathogenesis of AD.

In addition, defects in genes like the DNM1L and MFF, which mediate mitochondrial fission, have been associated with diseases characterized by abnormal mitochondrial morphology and function (15).

PBM restores mitochondrial health, which is critical for dealing with this genetic modification (16, 17). The effect of PBM on mitochondrial function is further elaborated in the relevant sections to follow.

PBM review studies have touched on the genetics of AD (18–20) but it is apparent that PBM does not directly mitigate the genetic risks associated with the onset of AD or alter the disease’s progression at the genetic level. However, evidence shows that PBM can alleviate AD symptoms and provide temporary cognitive improvements without reversing the underlying genetic factors. For example, oxidative stress can damage nuclear DNA (21), which may be addressed by the PBM’s ability to reduce oxidative stress (22).

Saltmarche et al.’s 2017 study provided valuable insights into PBM’s therapeutic potential, observing notable cognitive enhancements in dementia patients after 12 weeks of treatment (810 nm wavelength pulsing at 10 Hz). However, this was not sustained after treatment, and the patients rapidly resumed progressive decline (23). This pattern was echoed in a study on another progressive neurodegenerative disease, chronic traumatic encephalopathy (CTE), which is related to repetitive traumatic brain injury (TBI), in which patients experienced symptom relief during PBM treatment (810 nm wavelength pulsing at 40 Hz) that receded once the treatment ceased but improved again with resumed therapy (24).

These observations underscore that although PBM may offer symptomatic relief in progressive diseases like dementia, its effects appear to be temporary, necessitating ongoing treatment to sustain improvements. Genetic predispositions, as evidenced by the presence of specific alleles like APOE ε4, seemingly resume their influence on disease pathology once PBM treatment is discontinued. Based on these small studies, the findings highlight PBM’s role in managing downstream pathophysiology symptoms rather than altering the genetic trajectory of AD. They also point to the necessity for regular, possibly long-term, PBM treatment to maintain its physiological benefits.

### Environmental factors

Environmental factors form part of the complex pathophysiological landscape of AD. Studies have shown that exposure to environmental toxins, such as heavy metals, air pollution, and industrial chemicals, can contribute to the risk of developing AD. Chronic exposure to biotoxins produced by bacteria, molds, and viruses can lead to cognitive decline and the pathophysiology of AD (25). Heavy metals like lead, cadmium, and manganese are known neurotoxicant that have also been linked to AD pathologies (26). Environmental contaminants identified in postmortem tissues of patients with AD include pesticides, flame retardants, plastics, and industrial pollutants (27). Exposure to cigarette smoke, polluted air, and pesticides negatively impacts brain health and increases the risk of AD (28).

Shortly after the Gulf War in 1990–1991, service men and women began reporting multiple symptoms ranging from persistent headaches, widespread pain, chronic fatigue, cognitive dysfunction, mood dysregulation, gastrointestinal issues, skin abnormalities, and respiratory problems. This condition is known as Gulf War Illness (GWI), and it is caused by neurotoxic exposure leading to glutamate excitotoxicity (29). In a blinded, randomized study of 48 patients with GWI, PBM produced increased concentration, relaxation, and sleep (30). Improved GWI outcomes with PBM therapy were also shown in a smaller case series (31). These GWI cases have shown that PBM can reduce the effects of environmental neurotoxins that might have contributed to AD.

Externally caused TBI as an environmental factor has been found to be a potential risk factor for AD or intensifying the risk (32). Over the years, research has shown that the mechanisms of PBM can alleviate the complex pathophysiological aspects of TBI, leading to positive outcomes in clinical studies (8).

### Infections

There is increasing suggestion of a potential link between infections and AD pathology. Specific pathogens, such as herpes simplex virus (HSV-1) and periodontal pathogens (33), have been implicated in AD development (34).

A meta-analysis to address the relationship between bacterial infection and AD found over a 10-fold increased occurrence of AD when there is detectable evidence of spirochetal infection (35).

During infections, Aβ functions as an antimicrobial peptide in the innate immune defense within the brain. It could play a role in the brain’s defense against pathogens. However, the burden of chronic infections can increase the risk of Aβ misfolding, potentially contributing to AD pathology. This implies that addressing infections is an important factor in AD therapy (36, 37).

There is no clear evidence that PBM can directly treat an infection as a drug. However, there is evidence that PBM can alleviate the symptoms of infection because it can treat the pathophysiology by boosting the immune system. This was the findings of a large-scale controlled clinical trial on the effects of PBM against COVID-19 (38). It is believed that the success of this treatment is attributable to the PBM’s ability to support the immune system to defend against infection, reduce inflammation, and accelerate wound healing.

### Blood–brain barrier integrity, cerebral blood flow, and vascular health

Blood–brain barrier (BBB) integrity, cerebral blood flow, and vascular health interact with each other and are discussed here.

#### Blood–brain barrier integrity and permeability

The integrity of the BBB plays a pivotal role in maintaining the brain’s microenvironment by shielding it from neurotoxins and pathogens. BBB dysfunction, characterized by increased permeability, represents an early development in AD pathology. This condition allows harmful substances to enter the brain, accelerating neurodegeneration.

Central to the BBB’s function is the neurovascular unit (NVU), which consists of neurons, astrocytes, endothelial cells that form the BBB, myocyte, pericytes, and the extracellular matrix (39). This unit orchestrates the complex interactions between the nervous and vascular systems, and it positions the BBB as an important intermediary. The overlapping pathophysiological characteristics of neurodegenerative and cerebrovascular diseases underscore the BBB’s vital importance. Consequently, strategies focused on preserving or restoring BBB integrity are recognized as promising approaches to slowing or reducing the severity of AD progression (40, 41).

#### PBM’s role in modulating BBB integrity

The potential of PBM as a therapeutic intervention to modulate BBB integrity has been a subject of increasing research interest. A pivotal animal model study conducted by Ganeshan et al. investigated the capacity of PBM to bolster BBB resilience against toxic insults, even when applied to body parts distant from the brain. The findings of this study show that PBM modulates molecular pathways within the brain’s vasculature, thereby reinforcing the BBB’s structural integrity (42). Specifically, their use of MPTP (1-methyl-4-phenyl-1,2,3,6-tetrahydropyridine) as a neurotoxin induced the generation of free radicals, simulating conditions of oxidative stress (43). The observed recuperation implies that PBM protects against neurotoxic substances and free radicals implicated in AD pathology, underscoring the BBB’s role in disease progression.

Contrarily, evidence from *in vitro* studies presents a complex picture showing that PBM can also increase BBB permeability. A study applying 808-nm PBM to BBB models demonstrated an increase in permeability (44). The immediacy of an *in vitro* process can be equated with a short-term process in the human body. These findings open possibilities for PBM to facilitate drug delivery across the BBB, aiding in the clearance of toxins and amyloid-beta deposits in a manner analogous to the glymphatic system’s function. However, this effect raises the possibility of increased vulnerability to external toxic insults during treatment windows. This seemingly dichotomous action of PBM—enhancing long-term BBB integrity while transiently increasing permeability during treatment—highlights the nuanced impact of PBM on BBB function.

Further complicating the PBM’s implications for BBB function in AD is its role in the synthesis of neuroprotective substances, such as glutathione. The antioxidant properties of glutathione (45), potentially synthesized in the liver (46), could be delivered more efficiently to the brain under conditions of increased BBB permeability induced by transcranial PBM.

In essence, PBM treatment has the potential to serve a dual purpose: safeguarding against the infiltration of neurotoxic agents over the long term and temporarily enhancing BBB permeability to facilitate the intake of beneficial substances and removal of harmful agents. This hypothesis, indicative of PBM’s intricate effects on BBB integrity and AD pathology, calls for further empirical investigation to validate its therapeutic efficacy and optimal application parameters.

#### Cerebral blood flow and vascular health

Reduced cerebral blood flow (CBF) is an early pathological mechanism in Alzheimer’s disease (AD), with significant evidence supporting its role in the disease’s progression. Chronic CBF reduction of 50% is associated with severe cognitive impairments. In humans, sustained CBF reductions exceeding 20% correlate with diminished attention capacity, whereas in rats, CBF reductions exceeding 30% adversely affect spatial memory (47, 48). These CBF reductions often result from the constriction of capillaries by contractile pericytes, a reaction potentially triggered by oligomeric Aβ. Such reductions are precursors to substantial neuronal damage and are linked to cognitive decline (49).

The transition from healthy aging to AD featured an accelerated decrease in CBF, ranging from 0.3–0.5% per year in healthy individuals to 2–5% per year in those diagnosed with AD. This fluctuation in CBF, both inter- and intra-individually, notably intensifies in AD, indicating a complex relationship between vascular dysfunction and AD pathology (50).

Additionally, there is a noted increase in atherosclerosis/arteriosclerosis within the intracranial arteries in AD, further complicating blood flow and underscoring the intricate link between vascular health, CBF, and neurodegenerative processes (51).

Given these insights, strategies that effectively enhance CBF could offer therapeutic benefits for AD. PBM stands out as a promising, noninvasive approach to mitigating vascular dysfunction in AD (52). Clinical studies and imaging with functional Magnetic Resonance Imaging (fMRI) have shown that PBM can significantly improve CBF and oxygenation levels in the brain, show improvements in cerebrovascular function following PBM treatment. These findings underscore PBM’s potential to reduce the risk of neuronal damage associated with AD progression.

#### Integrating BBB integrity, CBF, and vascular health

Over time, the compromised BBB facilitates the entry of neurotoxic proteins and other harmful substances into the brain, leading to neuroinflammation and impeding the clearance of Aβ, which contributes to plaque formation and neuronal damage. Furthermore, vascular abnormalities such as endothelial dysfunction, BBB breakdown, and cerebral microbleeds, are increasingly acknowledged not only as secondary issues but also as primary factors driving AD pathology. Collectively, these issues intensify a cycle of vascular dysfunction and neurodegeneration (53, 54).

Endothelial dysfunction, which is often a precursor to various vascular pathologies, impairs the production of nitric oxide (NO), which is essential for maintaining vascular health. This reduction in NO availability can lead to cerebral hypoperfusion, a state of reduced blood flow to the brain (55). Chronic cerebral hypoperfusion activates a cascade of negative outcomes, including oxidative stress, energy deficit, and further Aβ aggregation (56), underscoring the pivotal role of impaired CBF in AD progression. Additionally, reduced CBF can cause microvessel constriction and decrease their diameter, leading to further hypoperfusion and neurovascular dysfunction (57).

The presence of hemoglobin α (Hbα) in the blood vessel wall regulates NO delivery to vascular smooth muscle cells. A reduction or deletion of Hbα in endothelial cells can impair this delivery mechanism, thereby exacerbating vascular dysfunction (58). Thus, maintaining adequate hemoglobin levels, especially Hbα, is vital for preventing endothelial dysfunction and cerebral hypoperfusion, which are harmful to neurovascular function.

In this context, PBM has emerged as a promising noninvasive intervention for mitigating vascular dysfunction associated with AD. Yang et al. demonstrated that PBM preserved the expressions of Hbα and Hbβ that were significantly reduced in AD-afflicted rats, showing PBM’s capability to alleviate Aβ1-42 induced hypoxia in neuronal cells (59). This agrees with PBM’s role in preserving neuronal hemoglobin, which is essential for regulating neuronal oxygen homeostasis and supporting mitochondrial function in the brain.

Moreover, PBM induces NO release from endothelial cells and enhances the levels of angiogenic proteins, promoting angiogenesis. These actions address endothelial dysfunction and improve vascular health (60). A systematic review revealed that PBM can increase the expression of proteins associated with angiogenesis and reduce the activity of matrix metalloproteinase 2 and is associated with maintaining BBB integrity and vascular function. Among the reviewed studies, it was shown that intracoronary PBM irradiation can prevent restenosis, and PBM with a wavelength of 645 nm has been shown to stimulate angiogenesis (61).

By focusing on improving CBF, BBB integrity, and endothelial function, PBM is a novel approach to AD treatment that targets both neurodegenerative and vascular disease components, illustrating the multifaceted therapeutic potential of PBM in AD management.

#### Summary and implications

The relationship among BBB integrity, CBF, and vascular health plays a crucial role in the complexity of AD pathology, highlighting the need for comprehensive treatment approaches. PBM is a promising therapeutic strategy, potentially offering to preserve or improve BBB integrity, enhance CBF, and bolster vascular health. Despite these benefits, the nuanced impact of PBM on BBB functionality and its wider physiological effects require additional research. Further investigation is vital to clarify PBM’s true therapeutic potential and to determine the most effective treatment protocols specifically for AD.

### Gut-brain axis and microbiota

Recent research on the gut-brain axis has advanced our understanding of AD progression. The microbiota-gut-brain axis, a complex communication network linking the intestinal microbiota with the brain (62), could play an important role in the development and advancement of AD.

The gastrointestinal microbiota is known to affect the synthesis of various neurotransmitters and neural modulation compounds, thereby influencing brain function. Certain gastrointestinal bacteria can produce neurotransmitters such as glutamate, GABA, serotonin, and dopamine. These biochemicals are crucial to brain function and overall cognition (63).

Alterations within the microbiota-gut-brain axis are associated with the initiation and escalation of neuroinflammatory processes. These processes can release pro-inflammatory cytokines, ROS, and other neurotoxic molecules that contribute to neuronal damage (64).

Pathological changes in gut microbiota composition, often marked by increased gut barrier permeability and immune cell activation, are linked to compromised BBB function. This treatment can enhance neuroinflammation, promote neuronal loss, and thereby accelerate AD progression (65).

Studies have identified specific bacterial genera linked to AD pathology, suggesting a direct connection between gut bacterial products and the amyloid cascade in sporadic AD (66). Furthermore, taxonomic alterations in the gut microbiota present in AD offer new avenues for interventions targeting this axis (67).

Innovative treatments, including fecal microbiome transplants, have shown that modifying the gastrointestinal microbiota can improve outcomes (68). This approach opens therapeutic opportunities for PBM in treating AD and other neurodegenerative disorders because of its potential to modify the gut microbiota. Research using gut microbiota-targeted PBM in AD mouse models has yielded promising results (69). Administering PBM at specific red and NIR wavelengths directly to the abdomen improved cognitive function, reduced amyloidosis, and altered intestinal microbiota diversity, suggesting that PBM targeted to the gut may offer a non-invasive approach to modulating microbiota and mitigating AD pathology (69).

Studies on Parkinson’s disease (PD), particularly those delivering PBM to the abdomen, have provided insights relevant to AD. A 2019 review highlighted that PBM altered the microbiome toward a healthier composition in mice (70), and a subsequent human study observed microbiome changes in patients with PD following PBM treatment (71). Applying light on the abdomen directly exposes the microbiome to photonic energy, which could have a more immediate impact on bacterial cellular processes and signaling compared to transcranial irradiation.

In summary, the intricate relationship between the microbiota-gut-brain axis and AD underscores the relevance of this axis in AD development. Disruptions in this axis that lead to neuroinflammatory processes and neuronal damage are potential targets for PBM intervention. By targeting the gut microbiota, PBM has demonstrated promising outcomes in AD models, reinforcing its potential as a therapeutic modality for AD and highlighting the need for further research in this promising field.

### Glymphatic system

The glymphatic system is a crucial macroscopic waste clearance pathway in the central nervous system, leveraging a network of perivascular channels to facilitate the removal of soluble proteins and metabolites (72). Its significance in AD pathogenesis has become increasingly apparent, particularly in its role in clearing Aβ and tau proteins, which are hallmark markers of AD.

Recent research has revealed an association between glymphatic system dysfunction and the accumulation of Aβ, showing that impairments in glymphatic clearance mechanisms contribute to AD development (73). Aquaporin-4 (AQP4) water channels are instrumental in this system, aiding the clearance process. Disruptions in glymphatic function, often due to mislocalization of AQP4, have been linked to AD pathology, making AQP4 a potential target for therapeutic intervention (74).

The activity of the glymphatic system is notably enhanced during sleep, with a significant portion of waste clearance occurring in this state (75). The decline in glymphatic efficiency with age is correlated with sleep disturbances, indicating a potential connection between sleep quality, glymphatic function, and the progression of neurodegenerative disorders like AD. Thus, interventions targeting sleep and glymphatic flow may offer novel approaches for modifying disease trajectory in AD (76).

PBM has emerged as a promising modality for augmenting glymphatic flow and enhancing the clearance of brain waste, including Aβ aggregates. By potentially increasing AQP4 permeability and stimulating cerebrospinal fluid flow, PBM can facilitate the removal of neurotoxic aggregates, thereby reducing AD progression. Moreover, the application of PBM during nocturnal hours may improve sleep quality, further supporting glymphatic function and offering a synergistic approach to managing AD pathology (75, 77).

Recent findings also show that PBM by possibly modulating brain activity at gamma frequencies, could activate peptides that upregulate glymphatic activity, enhancing amyloid clearance (78). Given that transcranial PBM has been shown to affect the AQP4 channel for glymphatic flow and can train gamma waves in the brain at 40 Hz [along with alpha (8–12 Hz) and beta (13–30 Hz)] (79), points to PBM’s potential utility in improving glymphatic function.

In summary, the glymphatic system’s role in removing Aβ and tau proteins underscores its role in AD pathogenesis. Emerging evidence show that PBM enhances glymphatic function and waste clearance, showing a novel therapeutic strategy for AD progression. By potentially improving AQP4 channel permeability and sleep quality, PBM is a promising adjunctive treatment to bolster glymphatic efficiency and mitigate the impact of AD.

### Metabolic syndrome, insulin resistance, and dyslipidemia

Insulin resistance affects the brain’s glucose utilization, leading to energy deficits in neuronal cells, which may increase the risk of neurodegenerative diseases. Metabolic syndrome, characterized by insulin resistance, is significantly associated with a heightened risk of AD (80). This condition, sometimes referred to as “type 3 diabetes,” underscores the critical role of impaired glucose metabolism in AD development. Insulin is not only crucial for metabolic processes but also plays a significant role in learning and memory, showing that its dysregulation could contribute to cognitive decline (81).

Obesity, a key component of metabolic syndrome, induces chronic inflammation through increased ROS production due to lipotoxicity in adipose tissue. The inflammatory state exacerbated by obesity can damage cells and has been identified as a risk factor for AD (82).

Dyslipidemia, which is marked by abnormal blood lipid levels, such as high LDL cholesterol and triglycerides and low HDL cholesterol, is also a component of metabolic syndrome (83). This imbalance can lead to the conversion of systemic cholesterol to 27-hydroxycholesterol, which may cross the BBB, facilitating the deposition of Aβ and tau proteins, hallmarks of AD pathology (84). Dyslipidemia under pathological conditions may disrupt the BBB, alter the processing of amyloid precursor proteins (APP), and contribute to AD development (85).

PBM has been explored for its potential to address metabolic dysfunction. A previous study demonstrated that PBM can reduce blood glucose and insulin resistance and reverse metabolic abnormalities in skeletal muscle in diabetic mouse models (86). Improvements in insulin sensitivity and glucose metabolism, which are pivotal in metabolic syndrome, showing that improving insulin resistance and metabolic abnormalities could offer protective effects against AD.

Further research on obese and hyperglycemic mice treated with PBM showed significant reductions in body mass, blood glucose levels, and inflammatory infiltrates in abdominal adipose tissue after 4 weeks (87). These findings highlight PBM’s potential in mitigating dyslipidemia, further underscoring its potential role in reducing the risk or progression of AD.

In summary, the connection between metabolic syndrome, which is characterized by insulin resistance and dyslipidemia, and the increased risk of AD is becoming increasingly clear. PBM has shown promise in addressing these metabolic dysfunctions, potentially offering a non-invasive strategy to mitigate the risk factors associated with AD. By improving insulin sensitivity and glucose metabolism and reducing inflammation associated with dyslipidemia, PBM could play a role in preventing or slowing AD progression.

### Mitochondrial dysfunction

In our discussion of genetic factors, we explored how mutations in mtDNA lead to mitochondrial damage and dysfunction. This dysfunction contributes to cellular death and disease, including AD. Mitochondrial dysfunction affects mitochondrial bioenergetics, resulting in energy shortages within neurons, leading to AD pathogenesis. The critical involvement of mitochondria in AD pathology has led many researchers to consider the mitochondria-cascade hypothesis (MCH) as a key target for addressing the disease. MCH posits that deteriorating mitochondrial function triggers a series of downstream cellular changes characteristic of late-onset AD, encompassing Aβ amyloidosis, tau phosphorylation, oxidative stress, synaptic loss, and neurodegeneration (88).

Further discussion of pathophysiological factors such as Aβ plaques, neurofibrillary tangles (NFT), neuroinflammation, oxidative stress, energy deficit, and growth factor dysregulation highlights their connections to mitochondrial dysfunction. These reviews underscore the interconnectedness of these aspects with mitochondrial health, emphasizing the fundamental role of mitochondria in the broader landscape of AD pathology.

### Amyloid-beta plaques

The formation of Aβ plaques is a hallmark of AD pathology and is characterized by the abnormal accumulation of Aβ peptides in the brain (89). These peptides, derived from the cleavage of amyloid precursor protein (APP), are closely associated with the genetics and pathogenesis of AD (90). The enzymatic actions of beta and gamma secretases on APP produce Aβ fragments, which then aggregate into insoluble plaques, disrupting cellular function and contributing to neuronal damage and death (91, 92).

A pivotal controlled study in 2011 on presymptomatic transgenic mice presenting an Aβ protein precursor demonstrated that PBM significantly reduced the count of Aβ plaques, as observed in plasma samples. PBM treatment reduced the behavioral symptoms associated with advanced amyloid deposition and decreased the expression of inflammatory markers. Furthermore, it resulted in enhanced ATP levels, improved mitochondrial function, and increased c-fos expression, indicating an overall enhancement of neurological function (93).

Subsequent research further validated PBM’s efficacy in reducing Aβ deposition in the cerebellar cortex and NFT in the cerebral cortex and cerebellum of transgenic mice after a month of treatment (94).

PBM has been shown to influence the activity of microglia, among the brain’s primary immune cells, by downregulating Ca^2+^ and promoting microglial polarization toward an M2 phenotype. This polarization enhances the phagocytosis of Aβ plaques and modulates the expression of anti-inflammatory and inflammatory factors (95). The M2 microglia phenotype decreases an enzyme associated with the production of ROS, thereby reducing neuronal death (96). A previous study remarkably revealed that light flickering at 40 Hz could activate microglia to significantly reduce the Aβ burden in the visual cortex (97). Adding sound resonating at 40 Hz reduces Aβ burden in the auditory cortex and other parts of the brain (98).

However, a subsequent replicating study challenged these findings (99). Another study found that 10 Hz of PBM was more effective than 40 Hz in activating M2 microglia and clearing Aβ plaques (100). This suggests a reconsideration of whether 40 Hz is best for performing these functions or whether a combination of these or other frequencies would be better. This is further critically analyzed in a later section on *Aberrated Brain Waveforms Expression, Gamma*, and on proposed PBM parameter settings in *Considerations for PBM parameters to Maximize Potential Outcomes for Alzheimer’s, Pulse rate of 40 Hz, 10 Hz and 0 Hz (continuous).*

Studies propose that PBM could directly or indirectly affect the aggregation state of Aβ. Direct irradiation with red light that been observed to reduce Aβ aggregation (101). It has been suggested that a photoelectric coupling effect alters protein structures, diminishing Aβ aggregation and toxicity, and that NIR light is capable of disassembling Aβ in vivo (102).

Moreover, PBM may facilitate the reduction of Aβ deposits by enhancing clearance mechanisms, notably through the activation of the glymphatic system (77)—a component of AD pathogenesis discussed in the *Glymphatic System* section.

The dysfunctional BBB, which impedes the clearance of Aβ from the brain, is directly implicated in AD development (103). The role of the BBB in AD pathology and PBM’s potential to support BBB functions are elaborated in the section on *Blood–Brain Barrier Integrity, Cerebral Blood Flow, and Vascular Health*.

Despite regulatory directions, there are ongoing debates and controversies surrounding the efficacy of targeting Aβ in AD treatment, which underscore the usefulness of exploring beyond Aβ and tau aggregation for therapeutic strategies. PBM stands out as a promising single intervention capable of addressing the complex pathophysiology of AD through its multifaceted actions. The potential benefits of PBM as a comprehensive approach to AD treatment in these debates will be discussed in a later section, *PBM as a Multifaceted Approach to AD Pathophysiology Amidst Debates*.

### Neurofibrillary tangles

NFTs within neurons are another defining feature of AD pathology (104). These tangles are composed of the protein tau, which, in the context of AD, undergoes abnormal phosphorylation. This process causes tau to aggregate into tangles, disrupting the internal transport system of neurons and contributing to neuronal death (105). Given their role in AD pathology, tau-targeting therapies are considered promising for treating AD and related tauopathies (106, 107).

Research highlights the potential of PBM in addressing tau pathology. An animal study published in 2014 explored a model that developed NFTs and found that after four weeks of PBM treatment, there was a significant reduction in hyperphosphorylated tau, NFTs, and oxidative stress markers in the neocortex and hippocampus, approaching levels observed in wild-type (control) animals (108).

In support of this research, a 2022 study utilized AD-like transgenic rat models with marked increases in paired helical filament (PHF) intensity—an abnormal tau protein structure within NFTs—in both the cortex and hippocampus of AD rats compared with wild-type rats. PBM treatment significantly reduced elevated PHF intensity in AD rats, with no notable differences observed in wild-type rats treated with or without PBM (59).

Microtubules (MTs), which are essential for maintaining neuronal structure and function, are polymerized from tubulins. In AD, hyperphosphorylated tau loses its ability to bind to and stabilize MTs. PBM at an NIR wavelength of 810 nm pulsing at 10 Hz, to facilitate temporary transitions in tubulin structures, potentially enabling autophagy and the renewal of MTs (109).

In summary, the evidence underscores the potential of PBM in mitigating one of the key pathological markers of AD—NFTs. This reduction in NFTs appears to be closely linked to PBM’s capacity to diminish abnormal tau protein phosphorylation. Additionally, PBM’s influence on the structural dynamics of microtubules, which are crucial for neuronal integrity, further illustrates its therapeutic promise in the context of AD.

### Neuroinflammation

Neuroinflammation is a pivotal element in AD pathology, with the brain’s resident immune cells, microglia, playing a crucial role in this inflammatory process. These cells are activated by the presence of Aβ plaques and NFTs, and initially serve a protective role by attempting to resolve these pathologies. However, the transition from acute to chronic inflammation, sustained by ongoing activation of microglia and other immune cells, contributes significantly to neuronal damage and accelerates AD progression (110). This continuous immune response not only aggravates the accumulation of Aβ and tau protein but also propels the disease’s pathological processes further (111).

Recent research has broadened the perspective on inflammation’s role in AD, underscoring the importance of both localized neuroinflammation and systemic inflammation. Intriguingly, studies have linked systemic and intestinal inflammatory responses, integral to the gut-brain axis, with AD progression, suggesting a comprehensive inflammatory backdrop that spans beyond the central nervous system. These systemic inflammatory conditions have been implicated in intensifying neuroinflammation, promoting tau hyperphosphorylation, impairing Aβ clearance, and weakening the integrity of the BBB, all hallmarks of AD pathology (112).

PBM has been identified as a potential therapeutic approach for modulating intricate inflammatory processes in AD, addressing both brain-specific and systemic inflammation. In a notable study, daily whole-body PBM treatment using a 40-Hz gamma frequency flicker demonstrated anti-inflammatory effects in the brain and systemically in mice 10 days before an inflammatory challenge. This treatment regimen effectively downregulated proinflammatory cytokines linked to inflammasome activation, such as IL-1β and IL-18, and enhanced the levels of the anti-inflammatory cytokine IL-10 (113).

Further evidence supporting PBM’s anti-inflammatory capabilities comes from a systematic review of controlled animal studies, which confirmed PBM’s potential in mitigating neuroinflammation (114). Additionally, a literature review of PBM therapy’s efficacy in treating pain and inflammation highlighted its beneficial impact on proinflammatory biomarkers. These findings collectively highlight PBM’s capacity to attenuate inflammatory responses (115), showing its viability as a non-invasive treatment option for countering inflammation-associated pathological processes in AD.

In summary, the critical role of inflammation, both within the central nervous system and systemically, in driving the progression of AD from amyloid and tau pathology to cognitive decline has been increasingly recognized. Targeting these complex inflammatory pathways, PBM is a promising therapeutic strategy, offering potential benefits in reducing inflammation and thereby slowing AD neuropathological progression.

### Oxidative stress

Oxidative stress, a factor in AD development, arises from an imbalance between reactive ROS production and the body’s ability to neutralize its damaging effects. This imbalance is further exacerbated by mitochondrial dysfunction, which not only leads to diminished energy production but also escalates further oxidative stress, creating a self-reinforcing cycle of cellular damage (116). The cellular damage from mitochondrial dysfunction and oxidative stress is a driver in AD’s progression (117, 118). The process includes increased ROS production, oxidative damage to cellular lipids, proteins, and DNA, and reduced ATP generation efficiency. This scenario also underscores reduced energy metabolism, which is hampered by decreased activity of enzymes involved in oxidative phosphorylation (118).

Although mitochondrial dysfunction is a primary source of oxidative stress in AD, additional external factors, such as environmental pollutants, diet, metal exposure, and infections, also contribute to the oxidative burden (118, 119).

Although the metabolic activities of PBM and ATP production activate ROS in a transient manner, they alleviate oxidative stress by increasing mitochondrial membrane potential, restoring calcium homeostasis, activating synaptic signaling pathways, upregulating antioxidant enzymes, and improving overall mitochondrial function (17, 120, 121). Specific studies using hippocampal cell lines from sacrificed mice demonstrated that PBM enhances the activity of antioxidant enzymes in the hippocampus, effectively reducing ROS levels and oxidative stress there (122).

In summary, the reciprocal relationship between oxidative stress and mitochondrial dysfunction plays a significant role in AD pathogenesis, contributing to a cycle of chronic neuronal damage. Although PBM activates low-level transient ROS, it intervenes in this cycle by improving mitochondrial function, signaling pathways, restoring calcium homeostasis, and stimulating the brain’s antioxidant defenses. By directly diminishing oxidative stress, PBM is a potential therapeutic strategy for combating AD pathology.

### Calcium dysregulation

Calcium is a crucial cellular messenger that plays essential roles in synaptic transmission, neuronal excitability, and signal transduction. Mitochondrial dysfunction intensifies calcium mishandling, leading to calcium dysregulation in homeostasis. Disruption in normal calcium homeostasis can induce formation of Aβ plaques, accumulation of NFT, and dysfunction of synaptic plasticity, which, in turn, can affect calcium homeostasis, thereby forming a vicious cycle (123, 124).

Calcium ion (Ca^2+^) homeostasis and related mechanisms are responsible for learning and memory. In AD pathology, Aβ can disrupt Ca^2+^ signaling through several mechanisms with increased influx of Ca^2+^ from the extracellular space and dysregulating its release from intracellular stores (125). Intracellular calcium dysregulation may contribute to synaptic dysfunction, neurodegeneration, and cognitive impairment observed in AD (126).

A study found that 650 and 808 nm (not 1,064 nm) light promotes intracellular Ca^2+^ elevation. Ca^2+^ release from endoplasmic reticulum is activated by ROS generated by PBM (127), which hypothetically balances extracellular influx in AD. Balanced calcium influx supports neuronal activity and contributes to physiological processes essential for learning and memory (128). As an additional note, NIR light can influence calcium channels, leading to an increase in cytosolic calcium levels while decreasing mitochondrial calcium concentrations (127). The total effect of calcium dynamics on neuronal function and cellular homeostasis can be significant in the context of AD.

In summary, PBM’s capacity to adjust calcium dynamics reflects its therapeutic potential in restoring neuronal and cellular homeostasis affected by AD. This indicates that PBM can be a non-invasive strategy to mitigate calcium-related disruptions in synaptic function and neurodegeneration, providing a promising avenue for AD treatment and cognitive impairment mitigation.

### Reduced energy production and metabolism

Altered energy production and metabolism impact the development and progression of AD. Metabolic alterations in the aging brain and AD-related metabolic deficits are associated with glucose metabolism dysregulation, glycolysis dysfunction, the tricarboxylic acid (TCA) cycle, oxidative phosphorylation (OXPHOS) deficits, and pentose phosphate pathway impairment (129). These factors are intricately related to mitochondrial function (130).

Mitochondria play a crucial role in the production of adenosine triphosphate (ATP), the cell’s energy currency, which is essential for maintaining cellular functions, particularly in high-energy-demanding cells like neurons. Therefore, problems with mitochondria could be major contributors to AD pathology (131).

A decline in glucose availability in the brain compromises cellular respiration. This condition leads to metabolic stress that alters the processing of Aβ protein precursors associated with Aβ plaques and tau tangles, which are characteristic pathological features of the disease (130).

Patients with AD exhibit reduced levels of nicotinamide adenine dinucleotide (NAD) and altered mitochondrial metabolism. These inherent deficiencies in energy management may contribute to neural dysfunction and cellular degeneration, potentially leading to AD (132).

A systematic review showed that PBM can restore mitochondrial function (133). This is further elaborated in a previous section, *Mitochondrial Dysfunction*.

In summary, PBM can effectively restore mitochondrial function, enhance ATP production, and improve cellular energy management. By targeting the underlying mitochondrial and metabolic dysfunctions in AD, PBM is a promising therapeutic approach that can potentially slow the progression of AD.

### Precursors of AD symptoms

#### Neuronal, volumetric, and neurotrophic growth factor loss

As the disease progresses, there is a loss of neuronal volume, particularly in regions of the brain that are important for memory and cognitive function, such as the hippocampus and cerebral cortex. This neuronal loss results in brain matter atrophy and subsequent cognitive decline in AD. The severity of neuronal loss in the hippocampus is closely associated with AD (134). The resulting disruption in the vital processes in neurons, lead to widespread damage, loss of brain volume, and atrophy. As neurons stop functioning properly, they lose connections with other neurons, and eventually die. Patterns of cortical atrophy have been shown to accurately track disease progression and appear promising for the identification of AD subtypes (135).

AD affects the neurotrophin family of growth factors. The components include nerve growth factor (NGF), brain-derived neurotrophic factor (BDNF), neurotrophin 3 (NT-3) and neurotrophin 4/5 (NT-4/5) (136). NT-3 mRNA and protein levels remain unchanged in the AD brain, with minor reductions observed in NT-3 in specific brain regions like the motor cortex. Additionally, NT-4/5 levels are relatively stable in AD pathology. These neurotrophins play roles in preventing the death of specific neuronal populations and supporting neuronal survival (137). However, the main neurotrophic targets for AD therapy are NGF and BDNF.

Decreased NGF levels have been observed in the nucleus basalis of Meynert in patients with AD (138). The nucleus basalis of Meynert is a group of neurons in the basal forebrain rich in acetylcholine (Ach) and choline acetyltransferase (ChAT), which are known to undergo degeneration in AD. These reductions in NGF levels and dysfunction contribute to cholinergic degeneration, leading to cognitive dysfunction (139).

In AD brains, BDNF levels are decreased, indicating a lack of trophic support. This may contribute to disease progression. BDNF is a neurotrophin that plays an important role in regulating synaptic activity, neurotransmission, neuronal repair, and plasticity in the central nervous system. More specifically, BDNF is linked to learning and memory (140).

In summary, AD pathology involves alterations in neurotrophin levels, particularly decreases in NGF and BDNF levels, which are crucial for neuronal survival, cognitive function, and memory.

To counter neuronal loss and atrophy, PBM therapy stimulates the production of neurotrophic factors, such as BDNF and glial-derived neurotrophic factor (GDNF), which are essential for neuronal survival and function. More specifically, it rescues dendrite atrophy by upregulating BDNF expression (141). PBM also increases BDNF expression in the hippocampus (122).

As a demonstration of the PBM’s ability to harness NGF, an animal study showed that damaged peripheral nerves in the central nervous system could regenerate with NIR light irradiation (142).

The underlying mechanism of neuronal volume and growth factors may be PBM’s ability to enhance cell differentiation from stem or progenitor cells to near-target or target cells. The relevant cells are related to the central nervous system and peripheral sensory neural structures (143).

PBM might prevent synaptic loss caused by excitotoxicity associated with imbalanced neuroligin (Nlgn) proteins. Nlgn is a transmembrane cell adhesion protein localized at the postsynaptic membrane that contributes to the maintenance of synapses. A study demonstrated that PBM improves synapses and cognitive function and reduces epileptic seizures by inhibiting the downregulation of Nlgn3 (144).

In summary, PBM is a promising approach to address the neuronal loss, brain atrophy, and neurotrophic factor deficits associated with AD, with evidence in areas related to memory and cognition, such as the hippocampus and cerebral cortex. By stimulating the production of key neurotrophic factors like BDNF and GDNF, PBM supports neuronal survival, enhances synaptic plasticity, and fosters neural repair and regeneration. PBM not only promotes the upregulation of essential growth factors, it mitigates synaptic loss, and improves cognitive functions by counteracting excitotoxicity.

### Changes in brain networks and connectivity

In AD, changes in brain networks play a role in the progression of the condition.

The brain network tends to shift toward a more random structure as AD progresses. Alterations in network characteristics, particularly at the earliest stage of the disease, lead to changes in functional connectivity involving the somatomotor, frontoparietal, and default mode modules (145).

AD-related changes in brain networks are also widespread and extend beyond affecting memory and attention, affecting sensory and motor circuits. These changes challenge previous assumptions about AD’s effects and show that early-stage AD may exhibit broader cognitive impairment that is detectable during mild cognitive impairment (MCI) (146). These findings propose that the effect of AD is more global.

Different spatial brain networks are linked to different clinical phenotypes of AD, highlighting the diverse impact of AD on brain network modulation. The presence of misfolded protein aggregates characterizes neurodegenerative disorders like AD, leading to alterations in large-scale functional brain network organization, including language, dorsal attention, frontotemporal, and default mode networks (147).

The most widely studied resting state network is the default mode network (DMN), which has been associated with mind wandering, autobiographic memory, future thinking, and introspection (148). Its activity can reasonably be considered abnormal in AD and distinguishes AD from healthy aging (149).

Aβ accumulation disrupts DMN connectivity, even in cognitively healthy individuals, indicating an early involvement of the DMN in the disease process. Higher amyloid burden is associated with aberrant DMN connectivity and predicts longitudinal memory decline, pointing to a crucial link between amyloid deposition, DMN dysfunction, and cognitive decline (150).

PBM has shown promising effects on brain networks in the context of AD pathology in several ways by addressing the causes of network aberrance. It enhances neuronal activity, rebuilds damaged neuronal networks, and stimulating the production of neurons (151). It can effectively reduce amyloid plaque burden, improve mitochondrial function, increase ATP levels, and enhance cognitive function in animal models of AD. Moreover, it has demonstrated cognitive improvement, enhanced sleep quality, reduced behavioral symptoms, and increased brain perfusion in patients with mild to moderately severe dementia (19). It enhances synaptic connections, reduces synaptic degeneration, neuronal apoptosis, and neuroinflammation, and improves cognitive function in *in vitro* and *in vivo* models. Studies suggest that PBM modulates gene regulatory networks related to synaptic connections, with behavioral alterations, such as improved memory and reduced seizure scores, observed in PBM-treated mice (144). These effects lead to better brain networks.

Therefore, PBM is a promising tool to target the DMN in AD. Targeting DMN nodes, Saltmarche et al. studied 5 dementia patients with a PBM device that targeted the delivery of NIR light pulsed at an alpha frequency of 10 Hz to these nodes. They observed significant improvements over 12 weeks of treatment (23). In a later controlled study, Chao similarly targeted the DMN with PBM but delivered it at 40-Hz gamma frequency, on 8 patients (4 in active treatment, and 4 in usual care) over 12 weeks. Significant cognitive improvements were also observed (152), and fMRI revealed increased cerebral perfusion and increased connectivity between the posterior cingulate cortex and lateral parietal nodes within the DMN in the PBM group. This is elaborated further in the section, *Optimizing PBM Parameters for AD Treatment, Optimizing LED Brain Network Positioning,* to follow.

In summary, Brain network alterations are characteristics of AD. PBM addresses these brain network alterations by enhancing neuronal activity, reducing amyloid plaque burden, improving mitochondrial function, and increasing ATP levels, which collectively contribute to rebuilding damaged neuronal networks and stimulating neuronal production. These underlying mechanisms manifest as improved cognitive functions, enhanced sleep quality, and reduced behavioral symptoms, all supported by increased brain perfusion in patients with AD. They contribute to overall brain network health. PBM therapy, when targeted at the DMN nodes, brings these benefits to a network implicated in AD, achieving significant cognitive improvements in patients with AD. These findings underscore PBM’s potential as a non-invasive therapeutic strategy for addressing the complex changes in the DMN and other brain networks associated with AD.

### Aberrated brain waveforms expression

Connecting brainwaves or oscillations to AD is an emerging research area in AD pathology. Brainwaves are electrical patterns produced by the brain that can be measured by EEG. These brainwaves reflect the communication between neurons in the brain and are categorized by frequency and amplitude. There are five major types of brainwaves: 1. Delta Brainwaves: 0.5–4 Hz, 2. Theta Brainwaves: 4–8 Hz, 3. Alpha Brainwaves: 8–12 Hz, Beta Brainwaves: 12–30 Hz, Gamma Brainwaves: 30–100 Hz. These frequencies represent the speed at which brainwave patterns oscillate and are measured in cycles per second or Hertz (Hz). Each type of brainwave is associated with different mental states and activities, ranging from deep sleep and relaxation to heightened perception and problem-solving tasks. Brainwaves provide important insights into AD for early detection and therapeutic intervention.

### Gamma

The gamma frequency of 40 Hz has recently attracted significant attention. A pivotal 2016 study by researchers at the Massachusetts Institute of Technology (MIT) revealed that exposing AD mouse models to flickering light at this specific gamma frequency led to notable improvements in AD symptoms and markers. The findings revealed enhanced behavioral outcomes, activation of microglia and a substantial reduction in amyloid plaque accumulation within just 7 days, a remarkable finding at the time (97). Since then, the group has conducted a 3-month clinical trial on humans stimulated with light and sound at 40 Hz, but no significant change in cognition. However, there were improvements in ventricular size, stabilization of hippocampal size, and functional connectivity in both the DMN and medial visual network, as well as circadian rhythmicity after 3 months of stimulation (153). This area of research has settled into investigating audiovisual sensory stimulation (154) but not without detractors (99). In another smaller trial with only 40-Hz light flickering, there was no report of significant cognitive changes, but increased gamma neural activity was observed during stimulation (155). A similar device was later evaluated in a randomized controlled study involving 76 patients with mild to moderate Alzheimer’s Disease (AD), using an active-to-sham ratio of 2:1. The study successfully met its primary safety endpoint and demonstrated a 76-77% slower cognitive decline from baseline in the active treatment group compared to the sham group that was nominally significant (156). There is now a new clinical trial to follow this (157).

Gamma oscillations, which play a role in cognitive functions like memory, are disrupted in early AD, even before Aβ overproduction (158).

Murty et al. visually induced gamma oscillation in a large elderly population with mild cognitive impairment (MCI). The results demonstrated weaker gamma oscillations in individuals later diagnosed with early AD, particularly in the brain region responsible for processing visual images (159).

Based on these findings, restoring gamma oscillations is being explored as a potential therapeutic approach for AD, with the aim of improving cognitive function and intra-brain communication (160). However, some studies reported elevated gamma activity already present in patients with AD (161, 162), which cautions us not to be dogmatic about the need to increase gamma levels in AD brains. Gaubert et al. postulated that gamma power falls as the increase in amyloid burden level crosses a certain threshold level in later stages of AD. However, gamma power is high in preclinical AD, reflecting compensatory mechanisms (163). Based on the evidence, the gamma intervention should primarily be for achieving physiological outcomes, and whether it is present in patients with AD should be of little consequence. The patterns take care of themselves when their physiology improves.

A recent study revealed that shining light flickering gamma at 40 Hz activate the VIP peptide to drive the glymphatic system for better amyloid clearance, which contributes to improving AD physiology (78). This offers the glymphatic systm as an intervention target at 40 Hz.

#### Theta

Recent studies have reported the pivotal role of theta oscillations in memory recall, particularly in the context of AD. This body of research shows that targeting modifications of theta oscillations in specific brain regions could pave the way for novel therapeutic interventions. Elevated theta power within cortical areas has emerged as a potential biomarker of AD pathology. An investigation into the evolution of brain lesions and network alterations in AD—fueled by the presence of amyloid and tau biomarkers—upregulates theta power in the mid-frontal cortex, posterior cingulate cortex (PCC), and precuneus (Pc). This was coupled with a marked reduction in alpha band power in the posterior regions compared with amyloid-negative individuals. Over time, an increase in theta power, specifically in the PCC and Pc, was documented, indicating potential hypoactivation of the DMN in AD, thereby positioning theta as a marker for AD pathology (164).

Theta oscillations, described as traveling waves within the hippocampus, are believed to play a role in synchronizing phase coding for memory processes and spatial navigation uniformly across the hippocampus (165). Memory impairments have been linked to disturbances in both the amplitude and phase alignment of theta oscillations (ranging from 2 to 10 Hz) during the memory-encoding phase. The orchestration of theta oscillations across the hippocampal region by cholinergic circuits enhances memory. However, AD-related disruptions in these cholinergic circuits—located in the hippocampus—adversely affect memory through alterations in theta oscillations (166). Furthermore, altered hippocampal theta activity is associated with hyperglycemia, contributing to AD pathology (167).

Despite the contrasting characteristics of theta oscillations reported across different studies, it appears that in AD pathology, theta oscillations are pronounced in the cortical regions but are underrepresented in subcortical areas, especially in the hippocampus. A study focusing on young mice revealed that stimulus-evoked activity in the olfactory bulb (OB) enhances the oscillatory entrainment of the downstream lateral entorhinal cortex and hippocampus (168), providing a path to directly stimulate the hippocampus. By leveraging PBM and considering that tissues can resonate with pulsing frequencies when exposed to pulsed NIR light, it is hypothesized that NIR delivered to the OB could induce the desired oscillation in the hippocampus, such as at a theta frequency. This approach could potentially be implemented using an intranasal light-emitting diode (LED), as evidenced by the device used in the Zomorrodi et al. study, opening a new avenue for therapeutic interventions in AD (79). The targeting of the OB is further elaborated in the section, *Considerations for PBM parameters to Maximize Potential Outcomes for Alzheimer’s*.

#### Theta-gamma cross-frequency coupling

In AD, significant alterations in theta-gamma cross-frequency coupling (CFC) have been identified, highlighting its importance for cognitive functions, particularly in learning and memory. CFC could involve the nesting of higher frequency in the slow frequency such as gamma at 40 Hz inside theta at 4 Hz. Enhanced theta-gamma CFC within the hippocampus correlates with improved spatial learning and cognitive task performance (169, 170).

A previous study explored the connection between these deficits and theta-gamma coupling and revealed that individuals with AD showed the most significant working memory impairments and the lowest levels of theta-gamma coupling, particularly in challenging memory tasks, compared with healthy individuals with MCI (171).

Early-stage AD research, even before the onset of Aβ plaque accumulation, revealed a marked reduction in theta-gamma CFC in various mouse models of AD (172, 173). Bazzigaluppi et al. (174) observed that phase-amplitude coupling in the hippocampus and medial prefrontal cortex (mPFC) was impaired with decreased theta-gamma amplitude.

Similar impairments were noted in the parietal cortex, associated with spatial memory (175), and in the medial entorhinal cortex (176), suggesting a region-specific deficiency in hippocampal-prefrontal CFC as a functional marker of APP deficiency (175).

Tau accumulation in the entorhinal cortex increases theta-gamma coupling in the mPFC during associative learning in tau-expressing rats (177), indicating learning-associated neural oscillation disturbances in preclinical AD.

These findings underscore the potential of theta-gamma CFC alterations as early indicators of AD, which can be a target for intervention.

In summary, there is an opportunity to influence the theta-gamma CFC through PBM, as PBM has demonstrated its ability to alter brain oscillations. As previously discussed, employing a PBM with a 40-Hz setting and NIR light can upregulate endogenous gamma frequencies (79). This approach can be combined with a theta frequency ranging from 4 to 8 Hz, for instance, when delivered intranasally.

#### Alpha

Patients with dementia and mild cognitive impairment (MCI) exhibit reduced posterior alpha rhythms and impaired cholinergic basal forebrain tract function. Patients who respond to a drug treatment show more resilience to the reduction of posterior alpha rhythms (178).

A recent study found that 75% of MCI patients showed less abnormal alpha activity initially and a decrease in alpha activity after 6 months, independent of other AD biomarkers. This indicate that posterior EEG alpha decline correlates with MCI progression to AD (179).

Most early transcranial PBM studies were interested in general effects on brain function until Hamblin et al. (180) showed that pulsing at 10 Hz made a difference in TBI recovery in mice. Since then, animal studies and some human studies have examined the effects on both TBI (8) and AD pathologies. The application of 10 Hz was successfully performed for other conditions, indicating support for its outcomes for AD. Compared with continuous wave and 100 Hz waves delivered at 810 nm, 10 Hz waves were found to be most effective at improving neurological severity scores in mice with TBI (180). Another study using 10 Hz to treat mice with TBI showed improvements in learning and memory. These improvements are supported by significant increases in ATP and reductions in ROS when PBM is combined with metabolic agents like glucose, lactate, and pyruvate. It was also successfully tested for sleep deprivation (181).

The findings propose an opportunity for frequency-based interventions such as PBM targeting posterior regions, such as the visual cortex, in MCI patients to reduce the risk of AD progression. A PBM study on 5 dementia patients with various levels of cognitive impairment by Saltmarche et al. used NIR LEDs at 10 Hz and produced significantly better cognitive outcomes over 12 weeks of treatment (23).

The findings are supported by a mouse AD model study that demonstrated that cognitive and memory impairment can be improved using 1,070 nm NIR light delivered at 10 Hz to reduce cerebral Aβ burden. Their results show that the process triggered microglial responses in AD mice to reduce Aβ load and perivascular microglia to promote angiogenesis to further enhance Aβ clearance. Surprisingly, 40 Hz was also evaluated in this study, showing that 10 Hz was more effective than 40 Hz for clearing Aβ load, in contrast to other studies (100).

#### Summary of the PBM effect on brainwave aberrations

In summary, these reports favor the use of PBM in the treatment of AD regardless of the pulse frequency. It now leaves the matter of determining the correct parameters to increase efficacy odds and improve outcomes. The parameters that are most reported in studies on impact outcomes are pulse frequencies of 10 Hz (Alpha) and 40 Hz (Gamma) and wavelength of the light at 810 nm and 1,070 nm. An analysis of these findings can be used to contribute toward the development of a personalized set of parameters for individual patients with AD.

### Potential recovery from neurodegeneration through PBM neurogenesis

In the preceding sections, we explored a range of pathological factors contributing to neuronal death, as well as the potential therapeutic applications of targeting these factors using PBM. These interventions to prevent neuronal death can be deemed neuroprotective. In the following discussion, our attention will shift to neural regeneration or neurogenesis.

Evidence indicating a deceleration in the progression of dementia and potential signs of recovery indicates that PBM stimulates neurogenesis. The mechanisms underlying this phenomenon are proposed below.

Research indicates that PBM can stimulate the growth of neural stem and progenitor cells into neurons, enhance mitochondrial function to improve the neuronal environment, and reduce inflammation and gliosis, thus contributing to nerve regeneration. Specifically, PBM has been found to activate genetic and signaling pathways that support neuronal differentiation, facilitating the development of progenitor cells into functional neurons (151).

One area of focus has been the hippocampus, where PBM has been shown to improve the survival of new neurons by mitigating inflammasome and activating the AKT/GSK-3β/β-catenin pathway (182, 183). Studies have demonstrated that PBM enhances cognitive function and supports neural progenitor cell activities in models of brain injury and disease, including ischemic stroke and traumatic brain injury. For instance, transcranial PBM treatment for seven consecutive days after photothrombosis induction in a rat model reduced the infarcted area and increased the expression of neurogenesis and synaptic markers (184).

Chronic PBM has been shown to promote adult hippocampal neurogenesis by activating latent transforming growth factor β1 (TGFβ1) in an AD mouse model (185) and enhances the migration and differentiation of neural stem cells in the subventricular zone, a brain region prominently involved in neurogenesis (186).

In summary, PBM stimulates neurogenesis, which is a promising avenue for brain repair. While clinical evidence is encouraging, the studies are still in the early stages of statistical validation. For instance, the study by Chao et al., which was based on a single case of TBI, demonstrated neurogenesis through MRI findings, highlighting the potential of PBM in this area (187).

### Recovery from synaptic dysfunction and loss with synaptogenesis

Synaptic dysfunction is increasingly recognized as a pivotal factor in the pathogenesis of AD, alongside the formation of neuritic Aβ plaques and NFTs of hyperphosphorylated Tau protein (188). Notably, synaptic loss is considered the most significant predictor of cognitive decline in AD (189). The previous sections of this manuscript have explored various pathophysiological mechanisms contributing to AD that are indirectly and beneficially influenced by PBM. In this section, we delve deeper into the role of PBM in promoting synaptogenesis and protecting against synapse degeneration, highlighting its potential therapeutic implications for enhancing synaptic function in AD.

PBM shows significant potential in facilitating synaptogenesis, which is the process of forming synapses between neurons. It aids in the development of dendritic spines, encourages axonal growth, and establishes functional connections between new and existing neurons, contributing to the neural network’s structural and functional integrity (190). In temporal lobe epilepsy models, PBM has been observed to mitigate neurodegeneration and cognitive decline by upregulating synaptic-related proteins, such as neuroligin-3, indicating its role in enhancing synaptic connectivity and cognitive function (144).

Furthermore, PBM has been implicated in promoting functional recovery post-stroke by inhibiting neurotoxic markers, boosting synaptic expression, and modulating apoptotic pathways, thereby highlighting its neuroprotective capabilities (191). Additionally, its application in animal models of anxiety, depression, and AD has demonstrated alleviation of symptoms and neuronal cell death, further underscoring its therapeutic potential in neurodegenerative as well as psychiatric disorders by enhancing nerve fiber and synapse markers (192).

In addition to synaptogenesis and neurogenesis discussed above, PBM contributes to neural network formation by improving excitatory neurotransmission and oxidative metabolism, which are crucial for neuronal survival and differentiation. Long-term transcranial NIR irradiation has been shown to rejuvenate brain metabolism in aging rats to levels observed in younger rats, indicating its role in enhancing neural connectivity and overall brain health (193).

In summary, PBM has the capacity to support synaptogenesis in addition to neural network formation, indicating its further potential as a viable treatment option for AD.

### Summary and critical evaluation of PBM effects on AD pathophysiology and symptoms

This section provides a collective summary of the preceding findings and analyses, with a critical evaluation of the limitations. PBM offers a promising multifaceted approach to mitigating AD by targeting various pathophysiological factors. PBM has shown efficacy in addressing genetic predispositions, environmental factors, infections, BBB integrity, CBF, vascular health, the gut-brain axis, glymphatic system function, metabolic syndrome, mitochondrial dysfunction, and neuroinflammation.

By improving mitochondrial function, PBM enhances neuronal activity, reduces amyloid plaque and hyperphosphorylated tau burden, and increases ATP levels, contributing to cognitive improvements and synaptic plasticity. Additionally, it stimulates neurogenesis and synaptogenesis, potentially reversing neuronal loss and brain atrophy.

However, it is noteworthy that some findings are inferred rather than directly established. For instance, PBM’s modulation of microglial responses from pro-inflammatory M1 to anti-inflammatory M2 phenotypes suggests a potential for amyloid plaque clearance and reduced neuroinflammation, but this connection is inferred from changes in microglial activity rather than direct evidence of plaque reduction. Similarly, while PBM has been observed to decrease Aβ aggregates in cell models, its direct impact on amyloid pathology in human AD patients remains to be conclusively demonstrated. Furthermore, enhancements in mitochondrial metabolism and improvements in BBB integrity and vascular health through increased CBF are promising, but these effects need further validation in large-scale clinical trials. Current research is limited by small sample sizes, variability in study designs, and a lack of large-scale clinical trials to confirm efficacy and establish standardized treatment protocols, as will be presented in the following section, *Systemic review of the clinical evidence on PBM parameters in AD*. PBM’s long-term effects and optimal parameters remain under investigation.

While some causal links between certain pathophysiological aspects are still subject to more investigation, it is appropriate to emphasize evidence from human clinical studies, particularly controlled trials. The following sections of the manuscript will review and analyze this clinical evidence and propose potential treatment protocols based on brain networks and waveforms.

### Systematic review of the clinical evidence on PBM parameters in AD

This manuscript examined the effects of PBM therapy on AD’s multifaceted pathophysiological aspects, focusing predominantly on cell culture and animal studies. To assess PBM’s efficacy in humans, we conducted a systematic review for identifying the impact of parameters on clinical outcomes. This review focuses on available full-authored human clinical studies on PBM for AD published in peer-reviewed journals up to January 2014. The search was performed using the PubMed, Google Scholar, and Cochrane Library databases with keywords “photobiomodulation,” “Alzheimer’s Disease,” “dementia,” and “clinical studies.” This process identified 7 studies with varying quality and methodologies. We conducted a qualitative analysis of these studies to derive insightful data that may be used to improve the PBM treatment parameters. This systematic review was conducted following the PRISMA guidelines. The findings, along with the qualitative assessment of the included studies, are presented in a summarized table format for clear comparison and analysis. The findings are presented in Table 1.

**Table 1:** Summary of clinical studies on photobiomodulation in dementia and Alzheimer’s disease.

Authors	Saltmarche, et al. (23)	Berman, et al. (194)	Chao (152)	Salehpour, et al. (195)	Chan, et al. (196)	Nizamutdinov, et al. (197)	Razzaghi, et al. (198)
Publication date	2017	2017	2019	2019	2021	2021	2024
Type of Impairment	Mild to severe	Mild to moderate	Mild to Severe	Mild	Mild	Mild to moderate	Mild to Moderate
No. of patients							
Active	5	6	4	1	9	37	6
Control	0	3	4	0	9	20	7
Duration of study (weeks)	12	12	12	4	6 min.	8	12
Parameters							
Wavelength (nm)	810	1,060–1,080	810	810, 635	810	1,060–1,080	810
Power density (mW/cm2)	25–100	N/A	25–100	14–75	20	23.1	150
Beam spot size (cm2)	1	N/A	1	0.12–1	N/A	650	3.14
Pulse rate (Hz)	10	10	40	0, 10	0	0	40
Treatment time (minutes)	20	6	20	25	6	6	20
Energy density (J/cm^2^)	15–60	N/A	15–60	10.65–112	N/A	N/A	75
Total dose (J)	240	N/A	240	10.65–6,975	7	N/A	300
Cumulative dose	45–540	N/A	45–540	447–46,872	N/A	N/A	1800
LED positions	DMN, full head, intranasal	Eyes	DMN, intranasal	Entire head, Intranasal	Frontal lobe Forehead	Cranial, Eyes	DMN
Significant clinical outcomes (*p* < 0.050)	Cognition, Memory, Lifestyle	Cognition, Memory	Cognition, Memory	Cognition, Memory	Cognition, Memory	Sleep, Cognition, Memory	Disability improvement

N/A, Unstated or unavailable; MCI, Mild cognitive impairment; DMN, Default Mode Network nodes.

#### Synthesis of PBM therapy clinical evidence for AD

The systematic review identified seven clinical studies that exhibit a diverse range of research quality and methodologies. The studies ranged from single case reports to randomized controlled trials, including up to 57 participants, reflecting the variability in research contexts and scales at which these studies have been conducted.

The treatment parameters across the studies were notably heterogeneous, indicating the need for standardization to establish consistent and reliable treatment outcomes. Despite differences in wavelength, power density, and treatment duration, the studies universally reported a good safety profile, with no adverse events associated with PBM therapy being documented.

Clinically, significant improvements were observed across several domains, particularly cognition, memory, and lifestyle factors. The consistent observation of symptomatic improvement highlights the therapeutic potential of PBM for managing AD symptoms. However, given the variability in the parameters and scales of these studies, there is a pronounced call for larger-scale, well-controlled trials to substantiate the efficacy of PBM as a treatment for AD.

The variability in PBM parameters—from the wavelength of 810 to 1,080 nm and reported power densities ranging from 14 mW/cm2 to 150 mW/cm2—presents a challenge in defining an optimal treatment protocol. Despite this, the reported improvements in cognition and memory and even disability suggest the therapeutic benefits of PBM. However, the small sample sizes and, in some cases, the absence of control groups introduce potential biases. Larger, more rigorous studies are crucial to overcome these limitations and confirm PBM’s efficacy.

Considering these findings, PBM therapy can be a promising treatment option for AD. This approach shows potential for not only cognitive and memory improvement but also addressing broader disability factors associated with AD. Nonetheless, further research is imperative to define effective treatment parameters and confirm these preliminary findings. Such advancements could provide the possibility of extending similar therapeutic benefits to other neurological conditions like Traumatic Brain Injury (TBI), as indicated by concurrent research (8).

#### Optimizing PBM parameters for AD treatment

PBM clinical research for AD has reached a juncture where the need for parameter standardization to optimize therapeutic effects is evident, a sentiment echoed across several reviews (6, 199, 200). Presently, the critical parameters shaping PBM studies include: 1. Choice of wavelength: laser or LED; 2. Power levels and densities; 3. Duration of each treatment; 4. Dose/fluence; 5. Surface irradiance, which considers whether there is direct contact and the distance from the light source to the target; 6. Positioning of the light source relative to the skin surface; 7. Pulse rate or frequency and the corresponding duty cycle. The heterogeneity of these parameters has historically led to disparate results (201), raising concerns about the consistency (202) and reproducibility of PBM research outcomes (203). A deep dive into this complex issue extends beyond the scope of this study, as patient variability and the specific physiological state at the time of treatment must also be considered.

In response to these challenges, the author has advocated for the use of artificial intelligence (AI) to personalize PBM parameters, enhancing treatment efficacy for TBI (8) and, by extension, AD. In the future, AI could potentially model all the key parameters, optimizing treatments based on the latest knowledge. The effectiveness of AI processing will depend on the quality and comprehensiveness of the input variables.

Out of the critical parameters highlighted for AI processing or current use, the most productive applications are likely to revolve around wavelengths, pulse rates, and the strategic placement of light sources to engage cortical brain and olfactory networks. These aspects are expounded upon in the upcoming sections. Other important parameters, such as power levels and densities, dose and fluence, and surface irradiance, are considered but not included for further discussion in this manuscript.

A major weakness is the absence of reporting on the irradiance, and/or the distance between the LED and the traget surface in clinical studies. This is because power density is highly attenuated by the distance between the LED and the target surface. A review has suggested that the low fluence/dose found effective in successful *in vitro* studies may not reflect the much higher dose required for effective *in vivo* tissue irradiation (201). This highlights the need for more high-quality *in vivo* studies, which are currently being investigated by the author and research collaborators.

### NIR Wavelengths of around 810 and 1,060–1,080 nm

Historically, the use of low-level lasers of 808 nm and 1,064 nm wavelengths in transcranial PBM has been influenced by their demonstrated efficacy in enhancing neuronal viability and mitigating neurotoxicity, including in the context of AD and other neurological conditions such as strokes (4, 204). These wavelengths have been widely adopted because of their ability to modulate cerebral functions and reduce Aβ toxicity and have been proposed for their synergistic use (95).

More recent studies have shown that wavelengths in the 810 nm and 1,060–1,080 nm range with non-laser LEDs are effective for cognitive improvement and treating neurocognitive impairments, with benefits such as enhanced cerebral oxygenation and improved cognitive and behavioral functioning in conditions like dementia (102). In their review of related evidence, Heiskanen and Hamblin concluded that “the current total evidence appears to support the idea that PBM is not dependent on lasers or coherence” (205). The collective discussions present non-laser LEDs with these wavelengths as viable alternatives to 808 nm and 1,064 nm lasers. The discussion in this section will focus on the wavelengths agnostic of whether they are from laser or LED sources.

There is a debate over the level of tissue penetration and mitochondrial activation offered by each wavelength, with computer modeling providing support for both 1,064 nm in laser irradiation and 1,070 nm in LED (206) irradiation and 808 nm in laser irradiation or 810 nm in LED (207–209). These models differ in terms of their input variables and algorithms, which affect their respective penetration calculations.

Specifically, for intranasal delivery, computer modeling showed that 810 nm offered higher light deposition into the brain than 1,064 nm (210). Empirical studies on human forearms have shown that 1,064-nm lasers maintain increases in oxygenated hemoglobin and longer oxidized cytochrome c oxidase (CcO) (211). Moreover, previous research indicated that 810 nm elicits a higher level of mitochondrial activation than 1,050 nm, with both wavelengths enhancing mitochondrial activity (212).

Salehpour et al. proposed that light between 600 and 850 nm primarily targets the mitochondrial electron transport chain (ETC), boosting neuronal respiratory capacity, as exemplified by the significant enhancement of ATP production at 808 nm (213). Conversely, wavelengths from 1,000–1,350 nm (labeled as NIR II) (214) are thought to be absorbed by structured water clusters in heat/light-gated ion channels, like TRPV1 (215), increasing the vibrational energy that perturbs protein structures and modulates intracellular Ca^2+^ levels (4). This action augmented ETC activity, as evidenced by increased oxidation of CcO upon exposure to 1,064 nm light (216).

It is notable that wavelengths of 750 nm and 950 nm have been found to inhibit CcO activity, advising against their use in PBM for effective AD treatment (217, 218).

In conclusion, although both 808/810 and 1064/1060–1,080 nm groups of wavelengths show potential for scalp application in PBM, computational models indicate a preference for 810 nm for intranasal delivery. Based on these findings, particularly the merits of these two wavelength groups, combining them in an intervention could enhance outcomes. The author is actively engaged in co-investigations employing EEG and fMRI to study brain responses to these wavelengths, and forthcoming research is expected to provide further insights.

#### Pulse rate considerations at 40, 10, and 0 Hz (continuous).

The optimal pulse rate for PBM in AD treatment remains a topic of substantial research and discussion, with the literature predominantly endorsing gamma frequency, particularly 40 Hz, for enhancing memory functions, as discussed in in detail in a preceding section, *Aberrated Brain Waveforms Expression, Gamma* (97, 154). In summary, much of the research in gamma stimulation is done with flickering audiovisual sensory stimulation giving promising results particularly with mouse model with some contention in replication studies. They provide useful information for PBM which in contrast, directly activates neural tissue via direct light penetration into brain nuclei. Recent transcranial PBM research on AD explored the impact of 40-Hz gamma stimulation in both animal (219) and human subjects (6, 220).

Concurrent to the exploration of 40 Hz, PBM interventions at 10 Hz were also considered, as addressed in detail in the *Aberrated Brain Waveforms Expression, Alpha* section, and showed superior results to those of 40 Hz (100). Ongoing research continues to explore 40-Hz interventions with reported efficacy in both animal (219, 220) and human studies (152). However, based on the merits of 10 Hz and contrasting data for 40 Hz, we would advocate for the inclusion of 10 Hz alongside 40 Hz in treatment protocols.

Continuous wave (CW) interventions at 660 and 810 nm targeting the prefrontal region have demonstrated psychomotor and EEG network improvements, indicating that slower waveforms, such as delta, theta, and alpha waves, may correlate with improved performance (221). This highlights the potential benefits of enhancing delta waves, although current non-invasive EEG technology, which is limited to cortical areas, prevents confirmation of these effects in the hippocampus, where memory benefits are expected to occur in conjunction with gamma oscillations (174, 222).

In summary, given the current clinical evidence, PBM at frequencies of 10 and 40 Hz, as well as continuous waveforms, can all be effective in treating AD. A case could be made for other pulse frequencies. However, the balance of evidence is in favor of 10 Hz and 40 Hz. Additionally, there is an implication that combining 10 Hz with 40 Hz or coupling 40 Hz with a theta frequency like 4 Hz could yield enhanced therapeutic results in AD treatment. The merit of theta-gamma cross frequency coupling is discussed in the earlier section, *Aberrated Brain Waveform Expression, Theta-gamma Cross-frequency Coupling.*

#### Optimizing LED brain network positioning

The discussion on LED positioning in PBM for AD centers around targeting the Default Mode Network (DMN). The DMN, implicated in AD pathology, is composed of subdivisions, including the ventral medial prefrontal cortex (VMPFC), dorsal medial prefrontal cortex (DMPFC), posterior cingulate cortex (PCC), precuneus, including the lateral parietal cortex, and entorhinal cortex (223). Imaging studies showing lesions and amyloid accumulation in these regions, substantiate the DMN’s relevance to AD (150, 224, 225).

The specificity of LED placement for transcranial PBM treatment has been a subject of debate. Studies suggest that when NIR light is applied to a focal area of the head, such as the forehead or prefrontal cortex, the brain exhibits a broadly distributed response, as evidenced by blood oxygen level-dependent (BOLD) signals (226, 227), cerebral blood flow (CBF) (228, 229), and blood oxygenation (230) measurements. These findings indicate that brain reactions are not strictly localized to the focal point of light application.

Nevertheless, Nawashiro et al. reported that a localized response is observed when the treatment duration is short, before the rest of the brain responds. Their BOLD imaging revealed increased activity directly beneath the NIR light contact point (226). This localized response is particularly noteworthy, given that CcO, the primary light-absorbing chromophore in PBM (17), prompts a targeted increase in activity when NIR light is precisely directed at a specific brain region. This finding bolsters the rationale for strategically placing LEDs over the subdivisions of the DMN where lesions and amyloid deposits are concentrated to achieve more favorable outcomes in AD treatment.

**Figure 4:**
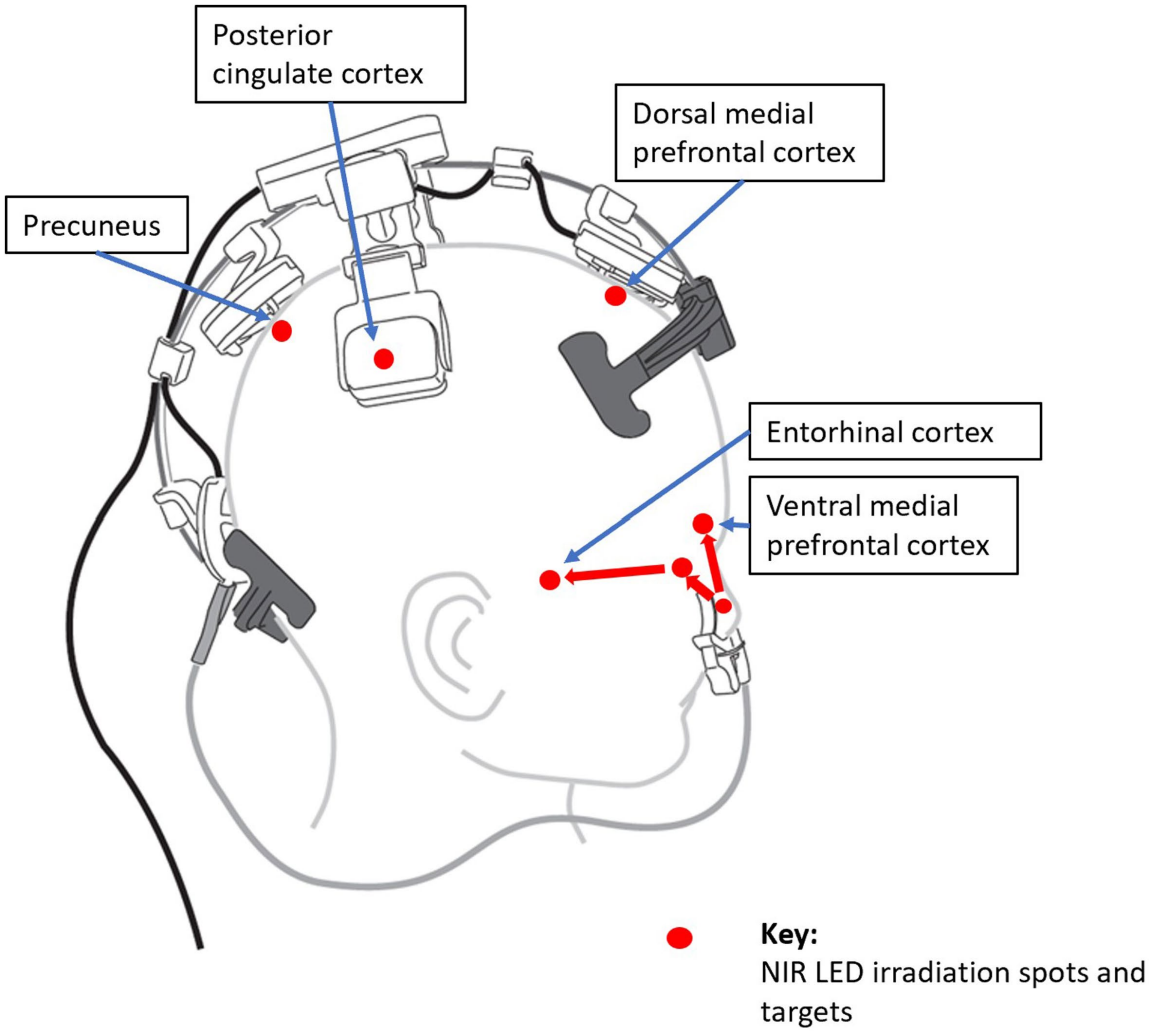
Strategic placements in the subdivisions of the default mode network. The precuneus, posterior cingulate cortex, dorsal medial prefrontal cortex, and ventral medial prefrontal cortex are accessed directly; while the Entorhinal cortex is accessed through the olfactory bulb, which is directly projected to each other (231). Image: courtesy of Vielight Inc.

For optimal AD pathology outcomes, positioning LEDs directly over the DMN subdivisions is likely to enhance the PBM effect. These positions are depicted in Figure 4, which illustrates how strategic placement can be designed.

#### Role of the olfactory bulb and neural network

Olfactory dysfunction serves as an early indicator of AD, with olfactory sensory neurons (OSNs) showing increased Aβ production and neuroinflammation, resulting in compromised olfactory capabilities. The olfactory neural network includes the entorhinal cortex (EC), which is a component of both the DMN and the primary olfactory cortex, implicating it in the memory dysfunctions characteristic of AD (231). Although the link between olfactory impairment and AD would make it a predictive marker, the precise mechanisms underlying this relationship and its prognostic reliability remain subjects of scientific debate (232). Nevertheless, the presence of tau and Aβ pathology in the olfactory bulbs (OB) of patients with AD, particularly in those with clinically manifested AD (233), underscores the potential benefits of targeting the OB in the treatment of AD.

Considering the EC’s involvement in the DMN, the OB’s connection to this region also plays a pivotal role in the DMN and memory functions, which are often impaired in AD. Thus, PBM directed at the OB can have a dual impact on the DMN and olfactory function. The EC’s connection to the hippocampus further reinforces this dual impact, which is critical for memory processes (234).

Intranasal PBM is a practical method to target the OB directly, allowing LED light to reach various brain regions, including the ventral medial prefrontal cortex, as part of the DMN (235). See Figure 4. Given that PBM has been proposed to reduce Aβ load, as discussed in the *Amyloid-Beta Plaques* section, applying PBM to the OB could provide a multi-faceted approach to AD therapy, addressing both the olfactory neural network and DMN and potentially enhancing overall treatment efficacy.

In the section, *Aberrated Brain Waveforms Expression, Theta-gamma Cross-frequency Coupling*, we propose in more detail, experimenting with inducing the theta wave intranasally to couple with a gamma wave for improved memory function.

#### General PBM parameters and new input warranted

The parameters of PBM treatment, such as power density, treatment duration, and dose, should align with those established for other neurological conditions while being fine-tuned for AD-specific pathophysiology to yield more effective outcomes. These parameters, along with others detailed in the author’s research on TBI (8), will contribute to AD treatment. Although studies have been published on the biophysics and mechanisms of key parameters (201, 236, 237), an updated discussion to include newly discovered nuances in pulse frequency and wavelength effects is warranted in another paper.

We discussed the merit of PBM in gut-brain axis and microbiota in an earlier section. Gut-directed PBM strategies could be added to the more widely discussed transcranial and intranasal PBM approaches in this paper.

### Limitations of PBM as a treatment for Alzheimer’s disease

The application of PBM for AD faces considerable challenges, not least of which is the shortfall in comprehensive controlled human trials essential for affirming PBM’s therapeutic efficacy. A primary impediment is the impracticality of rigorously testing each newly discovered set of PBM parameters for optimization—ranging from wavelengths to pulse frequencies—through large-scale randomized clinical trials. The logistical and financial constraints of extensive testing limit the evidence base to smaller studies and anecdotal experiences.

Compounding this challenge is the lack of standardized methodologies across studies, leading to inconsistent clinical outcomes and complicating efforts to aggregate data or define universally effective treatment protocols for AD.

An often overlooked but important factor in PBM is the effect of the distance between the LED and the target surface area. The power density emitted by the LED decreases exponentially with distance before reaching the target surface. Many PBM clinical studies report the LED’s output power density but not the actual surface irradiation. This omission raises questions about whether the observed efficacy is due to a placebo effect, especially in studies lacking control groups or objective measures. The notion that personalized PBM treatment protocols could enhance therapeutic outcomes is compelling but remains largely hypothetical. A relatively limited selection of peer-reviewed research and that findings are mostly early-stage findings support the hypothetical status of the protocols. In time, controlled clinical trials will confirm or reject the efficacy of the protocols. However, due to the rapid self-learning nature of AI, it could be a tool that could help to overcome this limitation. For now, there is a significant gap in fully understanding PBM’s potential in treating AD, and the course suggested in this manuscript are just promising early steps.

### PBM as a multifaceted approach to AD pathophysiology

The understanding of AD pathophysiology, as discussed in this study, draws extensively from existing literature. However, this representation is far from exhaustive, and ongoing debates within the scientific community continue to shape our knowledge. Although current regulatory approaches tend to align with the Amyloid Cascade Hypothesis, with a focus on Aβ as a primary target, this stance is not without controversies. For instance, the initial identification of the Aβ molecule, specifically Aβ-56, as reported in Lesné et al.’s 2006 Nature publication (238), has been scrutinized for alleged image alterations (239). Despite these controversies, the clinical findings that support an increase in cerebrospinal fluid (CSF) Aβ1-42, and in plasma Aβ42/40 ratios, alongside decreases in CSF P-Tau, CSF T-Tau, and plasma p-tau181, have been interpreted by regulators and many researchers as indicative of the reduction of Aβ accumulation. This led to the regulatory approval of lecanemab and donanemab for market release (240, 241), albeit with consideration of potential side effects in ARIA (2).

Some researchers offer alternative perspectives on AD pathology; for instance, Weaver et al. introduced a novel concept that views AD as an autoimmune condition (AD2). This model proposes that Aβ functions as an early responder cytokine with antimicrobial traits, unintentionally targeting neurons because of their similar electrophysiological properties to bacteria. This phenomenon initiates a continuous cycle of neurodegeneration influenced by amino acid metabolism, thereby questioning the conventional Amyloid Cascade Hypothesis (242). In this context, PBM is an effective treatment for autoimmune disorders, an example being Hashimoto’s thyroiditis (243).

Upon reviewing the broader landscape of AD pathophysiology, it becomes evident that the complexity of the disease encompasses far more than the aspects highlighted by the Amyloid Cascade Hypothesis. Many researchers have argued for the critical importance of other pathophysiological elements, advocating for a treatment approach that addresses the different factors or facets of AD pathophysiology highlighted in the various sections of this manuscript. In this context, PBM is a promising, unique intervention capable of addressing various pathophysiological aspects. The potential lies in its ability to modify several underlying mechanisms of AD simultaneously, and importantly, with an established safety record (244).

However, the exploration of PBM as a therapeutic option for AD remains limited, largely due to insufficient engagement from the research community. This lack of investigation into PBM’s full potential of PBMs is attributed to funding challenges rooted in the difficulty of patenting natural light, which diminishes the incentive for significant private research investment. Despite these hurdles, the versatility of PBM as a non-invasive, multifaceted intervention underscores its potential significance in the evolving landscape of AD treatment strategies, warranting further exploration and validation within the scientific and medical communities.

### Future of PBM research in Alzheimer’s disease

As the exploration of PBM as a potential treatment for AD continues to progress, our understanding of its mechanisms and outcomes is expanding. The continual evolution of research findings necessitates adaptive adjustments to identify the most effective PBM parameters. The challenge of validating each modification through large-scale clinical trials—potentially delaying the application of significant technological advancement—highlights the importance of credible, expedited research strategies. Interdisciplinary collaboration and innovative methodologies stand out as pivotal approaches to refine and validate PBM’s therapeutic application in AD, emphasizing personalized treatments that consider the distinct physiological profiles of patients.

Interdisciplinary research focuses on brain functions, neural networks, and the molecular and protein structures affected by PBM. A key area of investigation is how PBM’s variable pulse frequencies of PBMs influence the polymerization and depolymerization of tubulin into microtubules (245). Such mechanisms may critically impact the formation of NFTs, which are important for AD pathology. Additionally, examining PBM’s effects on cellular electrical field, biophotonic emission, and other mechanotransduction mechanisms offers insights into its potential to enhance cellular communication and health (236).

Incorporating artificial intelligence (AI) into PBM research can significantly accelerate the discovery and validation processes. AI’s ability to model and predict the effects of various PBM parameters can hasten breakthroughs. Moreover, encouraging inter-organizational collaboration can broaden the research ecosystem, facilitating a comprehensive examination of PBM capabilities.

In summary, advancing PBM efficacy in AD treatment requires a concerted effort that combines innovative discoveries, strategic AI utilization, and controlled trials for validation. This comprehensive strategy could pave the way for the establishment of PBM as a viable AD treatment option.

## Discussion and conclusion

This manuscript aimed to construct a comprehensive layered model of AD by reviewing the multiple aspects of its pathophysiology. Despite significant investments and extensive research, the complexity of AD pathophysiology remains a formidable challenge, particularly the influence of genetics on disease progression. Mitochondrial dysfunction, which is largely attributed to inherited genetics, ultimately cascades into amyloid and tau protein burdens, and the therapies explored in the pharmaceutical field, largely just delay the inevitable course of the disease.

However, the large pathophysiological model presented here has made it evident that AD pathology is not defined solely by amyloid and tau loads. A more immediate and practicable path seems to be PBM, which can address many pathophysiological factors of AD as a singular, convenient intervention with an excellent safety profile. PBM’s simplicity compared with biologics makes it an attractive research area for those seeking novel AD treatments with a promising chance of success. This model presents PBM as a single intervention with multiple pathways that can individually address the various facets of AD pathophysiology.

PBM has shown potential to halt or even reverse the progression of AD, as evidenced by improvements in cognitive impairment and memory across various published studies, as discussed in the above section on *Systematic Review of the Clinical Evidence on PBM Parameters in AD* along with the information in Table 1. While the findings are promising, the studies conducted thus far have been small and employed highly variable protocols. Therefore, these results need to be substantiated through larger, controlled studies. Experience can be drawn from a clinical study involving neurodegenerative diseases in patients with chronic traumatic encephalopathy (CTE) treated with PBM. The subjects showed significant recovery with treatment but exhibited degenerative progression after discontinuation of treatment (24). This was also similarly reported in a case series of dementia patients treated with PBM (23). The improvement resumed after treatment recurrence, indicating the need for continuous treatment due to the persistent nature of genetic factors. Fortunately, PBM devices are low-cost and designed for convenient home use, thereby facilitating lifelong treatment.

The field of PBM has faced challenges in gaining disciplined research attention, but this is changing with the accumulating body of scientific evidence. Comparatively, PBM has more established mechanisms of action than other non-invasive brain stimulation techniques, such as transcranial magnetic stimulation (TMS) and transcranial direct current stimulation (tDCS), but it lags in research volume (246), partially due to funding challenges and the non-proprietary nature of light, which discourage large investment into research and clinical trials.

Recent clinical studies, albeit small and varied, unanimously point to PBM’s safety and its fundamental mechanism of mitochondria activation which is arguably a primary source of PBM efficacy. Recent reviews and discoveries indicate that efficacy could be elevated by optimizing key parameters such as wavelength, power density, LED positioning, duration and pulse frequency. We analyzed the merits of wavelengths concentrated around 810 nm and 1,060–1,080 nm, finding further potential in combining these wavelengths for improved efficacy. Additionally, we examined the merits of pulse frequencies, particularly focusing on 10 Hz (alpha) and 40 Hz (gamma), and the potential benefits of combining these frequencies. However, while other key parameters like dose/fluence, power densities, and irradiation are also important, there are currently insufficient data to form strategies around them. Ongoing research is expected to provide the necessary data, which will be reviewed in future studies.

Moving forward, our understanding of brain waveforms, oscillations, and neural networks is proving to be significant for determining outcomes. Because these can be finely adjusted in PBM devices, there is potential for synergy with diagnostic imaging techniques like magnetic resonance imaging (MRI) and electroencephalography (EEG). For the now, EEG makes its case as the most convenient method to provide quality temporal and even spatial data to guide the development and delivery of specific parameters for optimum outcomes. The deployment of an AI-assisted feedback loop based on EEG data may be the most feasible path for personalized treatment.

In conclusion, this model of AD pathophysiology showcases the multifaceted nature of the disease and the potential of PBM as a therapy that can address many aspects in a single intervention. Although clinical evidence of its efficacy is still emerging, continued investment in PBM research holds the promise of optimizing its parameters for effective AD treatment. For now, EEG-based AI platforms appear to be the future of PBM and represent a viable option to more effectively treat AD.
